# Artificial intelligence enhanced sensors - enabling technologies to next-generation healthcare and biomedical platform

**DOI:** 10.1186/s42234-023-00118-1

**Published:** 2023-08-02

**Authors:** Chan Wang, Tianyiyi He, Hong Zhou, Zixuan Zhang, Chengkuo Lee

**Affiliations:** 1 Department of Electrical and Computer Engineering, National University of Singapore, 4 Engineering Drive 3, Singapore, 117576 Singapore; 2 Center for Intelligent Sensors and MEMS (CISM), National University of Singapore, 5 Engineering Drive 1, Singapore, 117608 Singapore; 3 NUS Suzhou Research Institute (NUSRI), Suzhou Industrial Park, Suzhou, 215123 China; 4 NUS Graduate School for Integrative Science and Engineering, National University of Singapore, Singapore, 117456 Singapore

**Keywords:** Healthcare, Artificial, Wearable sensors, Implantable sensors, Volatile organic compounds, Neural interfaces, Human-machine interfaces, Artificial intelligence of things

## Abstract

The fourth industrial revolution has led to the development and application of health monitoring sensors that are characterized by digitalization and intelligence. These sensors have extensive applications in medical care, personal health management, elderly care, sports, and other fields, providing people with more convenient and real-time health services. However, these sensors face limitations such as noise and drift, difficulty in extracting useful information from large amounts of data, and lack of feedback or control signals. The development of artificial intelligence has provided powerful tools and algorithms for data processing and analysis, enabling intelligent health monitoring, and achieving high-precision predictions and decisions. By integrating the Internet of Things, artificial intelligence, and health monitoring sensors, it becomes possible to realize a closed-loop system with the functions of real-time monitoring, data collection, online analysis, diagnosis, and treatment recommendations. This review focuses on the development of healthcare artificial sensors enhanced by intelligent technologies from the aspects of materials, device structure, system integration, and application scenarios. Specifically, this review first introduces the great advances in wearable sensors for monitoring respiration rate, heart rate, pulse, sweat, and tears; implantable sensors for cardiovascular care, nerve signal acquisition, and neurotransmitter monitoring; soft wearable electronics for precise therapy. Then, the recent advances in volatile organic compound detection are highlighted. Next, the current developments of human-machine interfaces, AI-enhanced multimode sensors, and AI-enhanced self-sustainable systems are reviewed. Last, a perspective on future directions for further research development is also provided. In summary, the fusion of artificial intelligence and artificial sensors will provide more intelligent, convenient, and secure services for next-generation healthcare and biomedical applications.

## Introduction

The development of the fourth industrial revolution (Industry 4.0) has driven the rapid development and application of health monitoring sensors (HMSs) characterized by digitalization and intelligence (Shi, Qiongfeng et al. 2020a; Yang et al. 2022c; Zhang et al. 2023). HMSs have extensive applications in medical care, personal health management, elderly care, sports, and other fields, providing people with more convenient and real-time health services (Cho et al. 2020; Zheng et al. 2020). Health monitoring sensors have undergone a long evolution. The earliest sensors for heart monitoring appeared in the 1950s and were subsequently widely used in the medical field (Browder 1956). The vigorous development of wearable technology has transformed the use of health monitoring sensors from clinical monitoring in hospitals to long-term care in homes, such as wearable blood glucose monitors (Jessica et al. 2020), sports sensors (Dai et al. 2023; Gao, S. et al. 2021a, 2021b; Zhang et al. 2022), and respiratory monitors. In the 21st century, the emergence of smartphones has enabled the visualization of monitoring data, which has gradually been used in smart homes (Shi et al. 2021), remote medical care (Wang, C. et al. 2021), smart cities (Huang et al. 2023; Zheng et al. 2022), and other areas, providing people with more convenient and efficient services.

Although significant progress has been made in many aspects, health monitoring sensors still face many limitations (Beardslee et al. 2020; Lee et al. 2021; Li, P. et al. 2021b; Mohankumar et al. 2021). First, sensors can be subject to noise and drift, which can cause fluctuations in the measurements they provide (Dong et al. 2021; Kai Dong et al. 2020; Takeda et al. 2014). Then, with the increasing availability of sensors, it has become easier and more cost-effective to collect large amounts of data (Dong and Wang 2021; Zhou and Chai 2020). A significant challenge arises in making sense of the vast amount of data generated by health monitoring sensors and extracting meaningful insights. For instance, in healthcare applications, wearable sensors can be used to monitor the health and activity levels of users. These sensors generate large amounts of data encompassing factors such as heart rate, blood pressure, and movement. Besides, multiple sensors are used to monitor various aspects of the target (Borchers and Pieler 2010; Dong et al. 2022). These sensors may be independent of each other (Poitras et al. 2019), meaning that they are not designed to work together or provide a unified view of the system being monitored. Moreover, traditional sensors are typically characterized as open-loop, lacking feedback or control signals from the system they monitor (Ellison et al. 2022). Consequently, while these sensors can gather measurements or observations, they are unable to directly impact the system's behavior. Such limitations hinder the advancement of conventional sensors toward more intelligent and responsive capabilities.

The widespread application of artificial intelligence (AI) is a notable feature of Industry 4.0 (Huang et al. 2023). The development history of AI can be traced back to the 1950s (Steels 2007). Early AI technologies relied mainly on symbolic logic and expert systems. Over time, AI technology has undergone multiple transformations and advancements. In the 1980s, machine learning began to emerge as the main branch of AI, enabling computers to learn patterns from data and make predictions and decisions (Langley, 1984). With the rapid development of computer technology, especially the emergence of cloud computing and big data technology, AI has encountered new development opportunities. Deep learning became the main technology in the AI field, enabling the processing of large amounts of data and achieving high-precision predictions and decisions by constructing multi-layer neural networks (LeCun et al. 2015). In the field of health monitoring sensors, the development of AI provides powerful tools and algorithms for data processing and analysis, which provides solutions for developing limitations faced by HMSs (Zhang et al. 2023). The data collected by HMSs can be processed and analyzed using AI algorithms, thereby achieving intelligent health monitoring (Gao, S. et al. 2021a). Meanwhile, machine learning algorithms enable mine potential health problems from big data, providing more accurate diagnoses and treatment plans for doctors and patients (Wen, F. et al. 2020a, 2020b, 2020c). Integrating Internet of Things (IoT) technology, AI technology and HMS, a close loop with real-time monitoring, data collection, online analysis and diagnosis and treatment recommendations can be realized. Furthermore, by adopting encryption, identity authentication, and other technologies, the security of health data and the privacy of patients also get ensured. AI plays a crucial role in the development of health monitoring sensors. AI-enhanced sensors will provide more intelligent, convenient, and secure services for healthcare and biomedical applications.

**Fig. 1:**
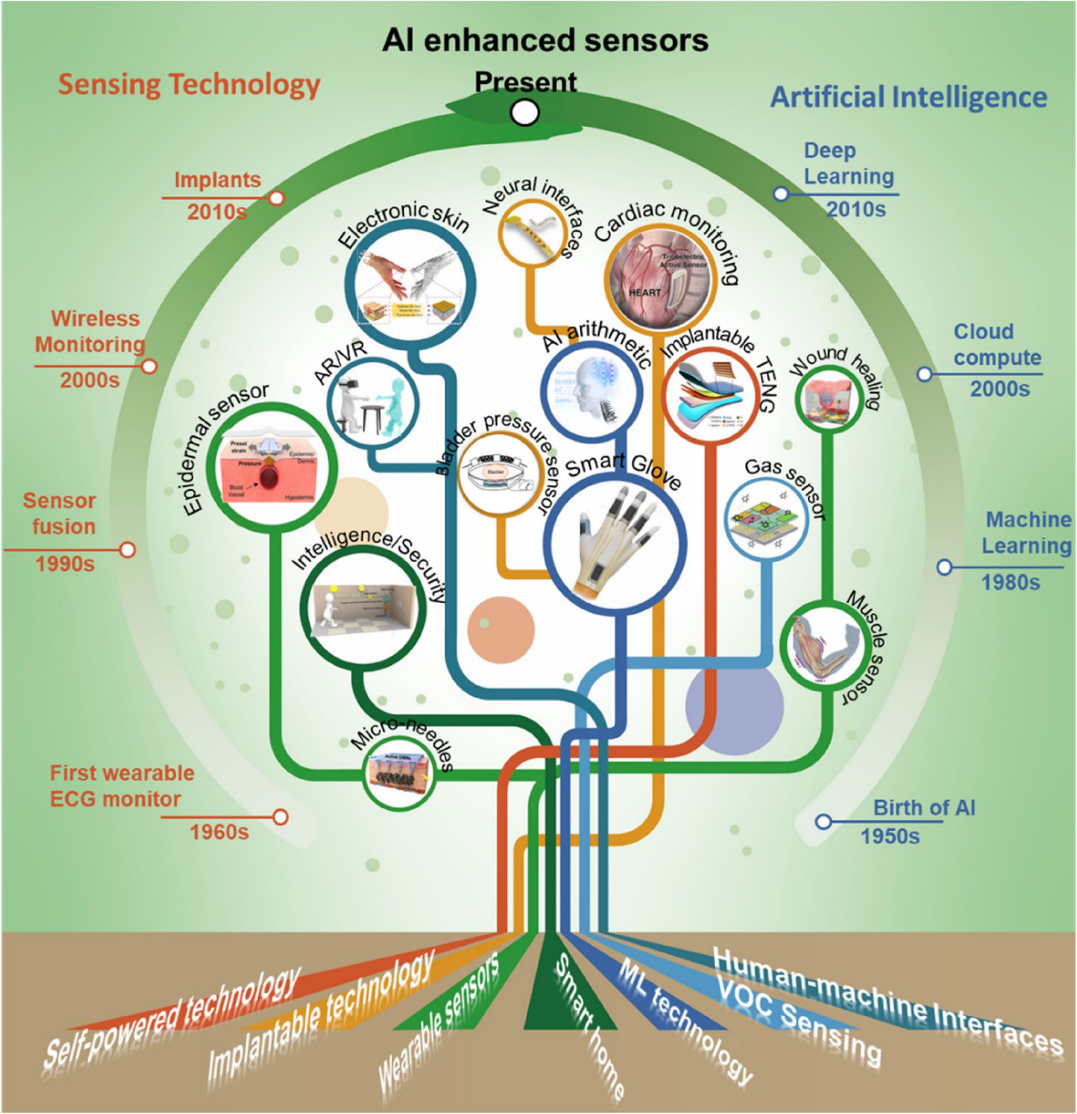
Overview of the development of artificial sensors enabled by intelligent technologies. This figure illustrates the road map of sensing technologies (left side) and artificial intelligence technologies (right side) and the development of diversified applications in self-powered, implantable, wearable sensors and smart home, machine learning (ML), VOC sensing, and human-machine interfaces

This review focuses on the development of intelligent technologies enhanced by artificial sensors (Fig. 1). In Section 2, we introduce wearable sensors for monitoring respiration rate, body motion, pulse, sweat, tears, etc. In Section 3, implants that can realize cardiovascular pressure detection, nerve signal acquisition, and neurotransmitter monitoring are introduced. In Section 4, flexible electronics for therapy are presented. In Section 5, sensors for volatile organic compounds (VOC) detection based on optical, and electrical principles as well as AI-enhanced VOC platform are reviewed. In Section 6, advances in human-machine interfaces are provided. In Section 7  "AI-enhanced multimode sensor", various multimode sensors which combine pressure, temperature, humidity, wireless transmission, and AI technologies are introduced. In Section 8, an AI-enhanced self-sustainable system is presented. Lastly, a short conclusion and perspective are provided in Section 9.

## Wearable sensors

### Wearable physical sensors

The continuous advancement of wearable electronics toward multifunctional wearable systems is driven by the desire to improve the quality of life through the enhancement of external device functionality (Ates et al. 2022; He et al. 2021; He and Lee 2021; Xu et al. 2019). Presently, commercially available wearable devices, such as wristbands, watches, and glasses, typically consist of rigid elements with flexible belts that are worn on the human body. However, to further optimize wearable comfort and enable advanced healthcare interactions with humans, wearable electronics are now progressing toward platforms with excellent flexibility, stretchability, and even self-healing capability, benefiting from significant advancements in the development of functional flexible materials (Liu et al. 2017; Wang and Urban 2020). Simultaneously, wearable sensors have made their way into the field of digital health, finding diverse applications in biomedical settings. These sensors enable the monitoring of vital signs (respiration rate, blood pressure, skin temperature, pulse, etc.) (Dias and Paulo Silva Cunha 2018; Iqbal et al. 2021; Wang, C. et al. 2018a), and physiological signals (electrocardiography (ECG), electromyography (EMG), electroencephalography (EEG), etc.) (Chen et al. 2020; Chi et al. 2010; Tian, L. et al. 2019a). They can also capture body kinetics such as strain and pressure (Bai et al. 2022; Gao, Shan et al. 2021a, 2021b; Zhang, Z. et al. 2020; Zhou, Z. et al. 2020a, 2020b), as well as dynamic biomolecular states through accessible biofluids like sweat (He et al. 2023; Wen, Feng et al. 2020a).

**Fig. 2:**
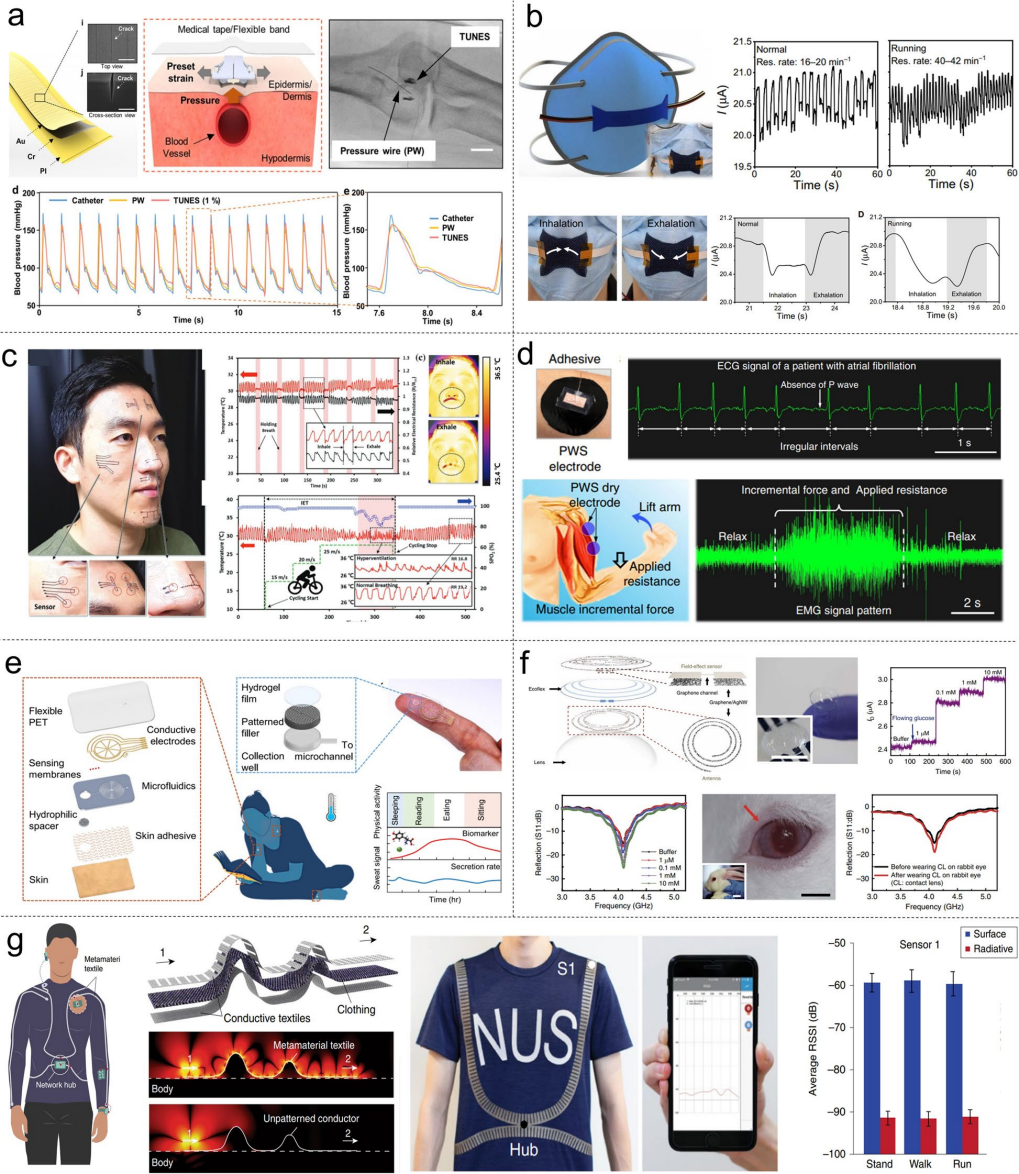
Wearable technologies for diversified applications. **a** A tunable, ultrasensitive, nature-inspired, epidermal sensor (TUNES) inspired by a spider’s sensory system with a tenable sensitivity by preset strain, which not only can measure respiration and muscle contraction but also serves as a skin-attachable biomedical sensor (Kim et al. 2023). **b** An respiratory rate sensor that detects breath patterns in real-time, which is fabricated by depositing PEDOT on a disposable mask (Clevenger et al. 2021). **c** A sensitive and flexible artificial skin comprising a negative temperature coefficient thermistor that can be attached to facial skin conformably, enabling the recording exhaled breath-induced temperature variations (Shin et al. 2020). **d** A highly conductive polymer dry electrode (PWS film) with excellent conductivity, stretchability, and self-adhesiveness, enabling high-quality ECG, EMG, and EEG signal acquisition in diverse conditions (Zhang, L. et al. 2020). **e** A wearable patch that enables continuous monitoring of the composition and rate of thermoregulatory sweat during periods of rest, which incorporates a fast sweat absorption mechanism and minimizes the time required for sweat accumulation, allowing for real-time measurement (Nyein et al. 2021). **f** A multifunctional contact lens sensor for wireless ocular diagnostics, which detects glucose in tears and intraocular pressure (Kim et al. 2017). **g** Interconnected wireless body sensor networks, characterized by energy efficiency and enhanced security, utilize radio surface plasmons propagating on metamaterial textiles (Tian, X. et al. 2019b)

Recently, Kim et al. developed a sensor called Tunable, Ultrasensitive, Nature-inspired, Epidermal Sensor (TUNES), which exhibits the capability to detect a broad range of signals, ranging from minute pulses to more substantial muscular contractions and respiration (Kim et al. 2023). As shown in Fig. 2a, the authors developed a sensor that mimics the geometry and tuning mechanism of the slit organ in spiders. By creating nanoscale cracks on a metalized polyimide (PI) film and adjusting the sensor's sensitivity through pre-strain, akin to a spider leg, they were able to measure strain and pressure across various scales using a single sensor. TUNES proves effective in measuring pulse waves at different pulse points and even during physical activities such as walking or cycling. To demonstrate the potential of the TUNES as a non-invasive biomedical sensor, a strain-controllable frame was designed to monitor diverse vital signals on the skin. To evaluate the sensor's accuracy and reliability, clinical trials were conducted, comparing its performance to that of a pressure wire that is widely regarded as the gold standard for intravascular blood measurement. The respiratory rate, which is an important physiological parameter, can be used to identify serious respiratory conditions such as cardiopulmonary arrest, chronic heart failure, or pneumonia. Figure 2b presents a respiration rate sensor fabricated on a face mask through oxidative chemical vapor deposition (oCVD), a unique method that can form patterned polymer films with adjustable thickness, enabling the sensor to maintain the advantageous features of fabrics (Clevenger et al. 2021). A highly conductive Poly(3,4-ethylenedioxythiophene) (PEDOT) layer has been successfully deposited on a disposable glove and mask, which functions as the resistive sensor to extract blood pressure information and respiratory rates in real-time with excellent precision. The sensor works by measuring the changes in current associated with the wearer's respiration, which detects inhalation and exhalation cycles as current drops and recoveries, respectively, and measures the duration and amount of current change for each cycle. By analyzing these changes, the sensor can estimate the wearer's respiratory rate and identify abnormal ranges, which could be indicative of various health conditions such as lung degradation, anxiety, fever, and cardiac conditions.

Temperature sensing plays a pivotal role in delivering critical data in various scientific and engineering domains. Recently, there has been a rising interest in wearable temperature sensors, driven by the need for flexible and sensitive temperature monitoring in applications such as artificial skin and continuous physiological temperature tracking. Figure 2c presents a new approach for creating highly sensitive and flexible artificial skin with a negative temperature coefficient (NTC) thermistor, which is made possible through a unique seamless structure and a monolithic laser-induced reductive sintering method (Shin et al. 2020). By utilizing heat-sensitive polymer substrates, it becomes possible to fabricate electronic skin with improved temperature sensitivity. The nickel oxide (NiO) temperature sensor can be conformably attached to various curvatures on the facial surface, enabling continuous and accurate measurement of physiological temperature over long periods. It has been demonstrated that it can capture small temperature variations associated with inhalation and exhalation. Besides, it was also able to accurately monitor changes in respiration patterns during intense physical activities. It revealed a notable 27% rise in respiratory rate and a significant 65% reduction in temperature variation amplitude, indicating shallow breath, when confronted with low blood oxygen saturation levels. These findings highlight the potential of it as a non-invasive physiological monitoring system, particularly for critically ill patients experiencing metabolic or pathologic dyshomeostasis conditions.

Accurate recording of human biopotential signals is crucial for diagnosing and treating heart, brain, and muscle-related diseases, which relies on the use of efficient wearable electrodes that interface effectively with the skin. As shown in Fig. 2d, Zhang et al. developed a highly conductive polymer dry electrode (PWS film), which possesses remarkable attributes such as self-adhesiveness, stretchability, and conductivity (Zhang, L. et al. 2020). It is achieved through solution processing of a blend comprising PEDOT: PSS, waterborne polyurethane (WPU), and D-sorbitol. The PWS film surpasses both conventional dry electrodes and gel electrodes in terms of lower skin-contact impedance and noise levels in static and dynamic measurements. Consequently, it enables the acquisition of high-quality signals for ECG, EMG, as well as EEG under diverse conditions. To evaluate the efficacy of PWS dry electrodes, a clinical study was conducted. This study involved the identification of electrocardiographic arrhythmia, detection of muscle activity during deep tendon reflex testing, and quantification of muscular strength during contraction and relaxation. The ECG results exhibited aberrant patterns consistent with atrial fibrillation, while the EMG signals obtained from PWS dry electrodes accurately captured changes in muscle activity during various tasks. These findings highlight the potential of PWS dry electrodes for clinical assessments of neurological conditions related to cardiac and muscular functions.

### Wearable chemical sensors

Obtaining a comprehensive assessment of an individual's health status requires collecting extensive information, including vital signs, physical activities, and chemical biomarkers from or near the human body. To address this need, wearable chemical sensors have been developed for the real-time detection of biomarkers from biofluids and the surrounding environment (He et al. 2023; Wen, Feng et al. 2020a). These sensors, utilizing different transduction mechanisms, can detect a wide range of biomarkers presented in biofluids such as tears or sweat, which could potentially enhance disease prediction, screening, diagnosis, and treatment. Figure 2e illustrates a recent study showcasing a wearable patch designed to continuously track sweat rate during rest periods (Nyein et al. 2021). To address the challenge of low sweat secretion rates during rest, microfluidics is integrated into the patch, which prevents evaporation and detects sweat rate selectively. Additionally, a laminated hydrophilic filler is incorporated, enabling rapid uptake of sweat into the sensing channel, hence reducing sweat accumulation time. The patch features functionalized electrochemical electrodes and electrical electrodes in the microchannels for simultaneous detection of pH, Cl^-1^, levodopa, and sweat rates. Due to its compact size, the device has been effectively employed in measuring sweat secretion rate under rest on different body parts, such as the finger, shoulder, and chest. The patch has undergone testing to evaluate its performance in dynamic sweat behaviors during light physical activities, glucose fluctuations, and drug administration for Parkinson's disease, demonstrating its potential as an ideal platform for daily monitoring of an individual's physiological status and medical condition in clinical and research settings. Tears and saliva offer several advantages over sweat when it comes to noninvasive health monitoring. These bodily fluids have a short accumulation time and provide sufficient volume, eliminating the need for additional extraction methods. Contact lenses have garnered significant attention as a viable substrate for tear sensors due to their biocompatibility and compliance. However, to address the potential risk of inflammation caused by wires in contact with the eyeballs, it is essential to establish wireless connectivity between contact lens-based tear sensors and the measuring equipment. For instance, as shown in Fig. 2f Kim et al. introduced a tear sensor using a graphene field-effect transistor (FET) integrated with a graphene-silver nanowire (AgNW) composite antenna, which works at radio frequency and wirelessly transmits sensory information upon the occurrence of glucose oxidation on the graphene channel (Kim et al. 2017). It is specifically designed to detect the level of glucose in tears and measures intraocular pressure by utilizing the resistance and capacitance properties. The real-time glucose detection in the tears of a rabbit eye and wireless tracking of intraocular pressure in a bovine eyeball in vitro have been successfully demonstrated.

### Wearable body sensors network

The integration of sensors, smart devices, and displays within the human body has been a significant development in wearable electronics research. However, establishing a functional network for these devices poses challenges, such as disruptions in physical activity caused by direct wiring between sensor nodes. Although wearable electronics have made progress in incorporating sensors and electrical wires into attire or on the skin, bridging them with other functional components that provide power and collect data remains challenging. Therefore, wireless interconnection approaches are crucial to create a network of discrete wearable devices, enabling seamless communication and functionality while preserving usability and user comfort. Wireless technologies, including radio-based methods like Bluetooth and Wi-Fi, are commonly used to connect wearable sensors for health monitoring and clinical notifications. However, these technologies require discrete power sources for each sensor node, facing issues of limited skin conformability, privacy concerns, and periodic battery replacement. Near-field communication (NFC) offers an alternative approach where sensors can be wirelessly powered by a reader, enabling battery-free and secure operation for various physiological measurements on the skin or inside the body (Lin et al. 2020; Lin et al. 2022; Niu et al. 2019; Shi et al. 2022). Figure 2g presents an innovative technique for constructing a wearable sensing system on textiles using metamaterial textiles to enable the propagation of radio surface plasmons on the body surface, leading to a secure and energy-efficient wireless body sensor network (Tian, X. et al. 2019b). The approach employs conductive fabrics in clothing that facilitate surface-plasmon-like modes at radio communication frequencies, resulting in body sensor networks with significantly higher transmission efficiencies than traditional radiative networks without metamaterial textiles. Moreover, wireless communication is limited to a range of 10 cm from the body, ensuring that sensing signals are secure and cannot be intercepted by external sources in the surrounding area. A network of two sensor nodes (Bluetooth modules on the shoulder and lower back) and a smartphone hub worn on the abdomen were built to demonstrate energy-efficient communication. Real-time detection of signal strength was conducted on a group of healthy volunteers during physical activity, and the results revealed a 31 dB enhancement in signal strength for both devices compared to the control group. The improved signal transmission efficiency by a factor of 1,000 results in reduced power usage and increased data transfer speed. These findings suggest that clothing can be used to manipulate electromagnetic waves around the body, providing a foundation for integrating concepts derived from microwave or photonic circuits into textiles, and enabling wireless sensing, signal processing, and energy transfer applications.

#### In vivo health monitoring

Compared with wearables sensors, which monitor body surface sweat markers (Gai et al. 2022), epidermal electrical signals (EMG, ECG, pulse) (Liu et al. 2022a, 2022b, 2022c, 2022d, 2022e; Ouyang et al. 2017), and body movements recanalization (Wang, C. et al. 2020a, 2022b, 2020c; Wang, C. et al. 2021), et al., implantable sensors function inside body perform more directly healthy states. For example, the normal functions of the heart and blood vessels (Ouyang et al. 2021), the recovery progress of the injured tissue (Zhang et al. 2021), and the abnormality of the central nervous signal (Shin et al. 2021), etc., all of which require devices to be implanted inside the body for a long-term status monitoring (Jiang et al. 2020). Meanwhile, the research on implantable devices also makes high requirements for materials selection, structure design, system integration, power supply, and biological safety (Jiang et al. 2018; Wang et al. 2019; Yao et al. 2022; Zhao et al. 2019).

### In vivo self-powered sensors

**Fig. 3:**
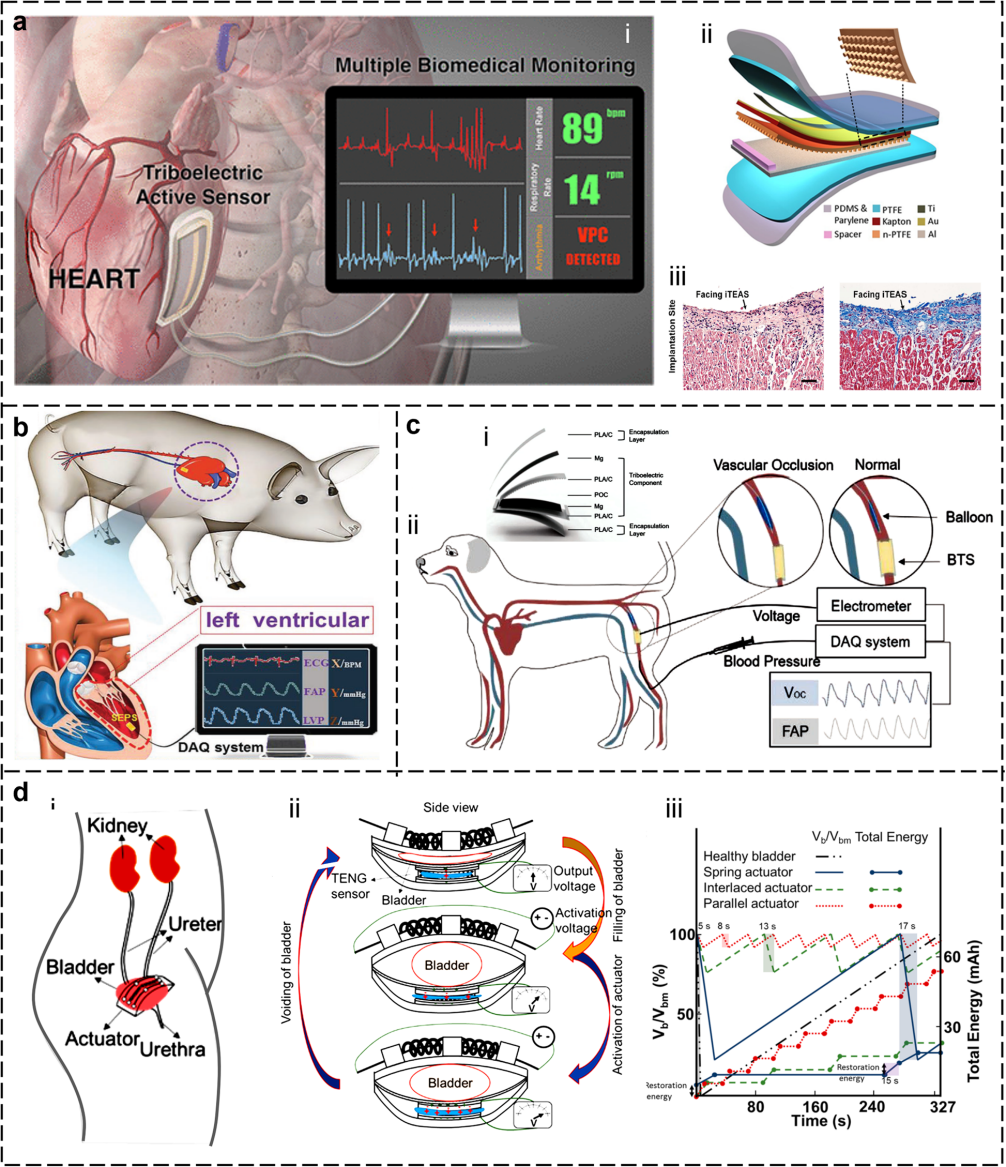
Self-powered sensor for in vivo healthcare. **a** A multifunctional triboelectric active sensor for accurate, continuous, and real-time monitoring of multiple physiological and pathological signs (Ma et al. 2016). **b** A miniaturized, flexible pressure sensor for intracardiac pressure monitoring, which can be combined with minimally invasive catheters (Liu et al. 2019). **c** A degradable and absorbable active pressure sensor for blood pressure monitoring and diagnosis of abnormal cardiovascular events (Ouyang et al. 2021). **d** A self-control system based on shape memory alloy for pressure monitoring and control of voluntary voiding of the neurogenic underactive bladder (Arab Hassani et al. 2018)

In recent years, the development of self-powered, implantable sensors exhibits the potential to revolutionize healthcare by providing continuous and accurate data without the need for external power sources or frequent replacements (Xiao et al. 2022; Yao et al. 2022; Zheng et al. 2020). Li et al reported the first self-powered implantable bioelectronics (SIBE) based on the principle of triboelectric nanogenerator (TENG) (Zheng et al. 2014). After that with the advantages of lightweight, low cost, and high output energy density, TENG-based SIBE stands out in vivo energy harvesting, cardiovascular monitoring, cardiac pacing, muscle/nerve electrical stimulation, tissue regeneration, and drug delivery. According to statistics from the World Health Organization (WHO), approximately 17.9 million people worldwide die from cardiovascular diseases (CVD) annually, accounting for 31% of global deaths. CVDs are also one of the main causes of disability (Ouyang et al. 2019; Shi, Y. et al 2020c; Wang et al. 2022). Ma et al. developed an implantable active pressure sensor, named iTEAS, for real-time biomedical monitoring, as shown in Fig. 3a (Ma et al. 2016). Based on the principle of triboelectric effect, iTEAS was able to convert mechanical signals generated by motion from the implant site into readable electrical signals for further analyzing heart rates, blood pressure, blood flow, and respiratory rates (Fig. 3ai). Simultaneously, iTEAS could harvest mechanical energy from the human body and convert it into electrical power to self-supply, overcoming the limitations of battery capacity (Fig. 3aii). After good packaging, this active sensor remained to monitor functions for 72 hours after the closure of the chest and behaved good biocompatibility (Fig. 3aiii). Figure 3b, (Liu et al. 2019) presents a novel approach to monitoring intracardiac pressure using a self-powered pressure sensor (SEPS) that is integrated with a minimally invasive implanted heart catheter. After optimization exploration, SEPS achieved a sensitivity of 1.195 mV/mmHg with a linearity of R^2^=0.997. The SEPS demonstrated its feasibility through in vitro and in vivo experiments, addressing the limitations of traditional invasive monitoring methods by providing a minimally invasive and self-powered solution.

Transient electrons can be degraded and absorbed within the body after they complete their service, saving patients from pain and the risk of infection from a second surgery to remove the implant (Chao et al. 2020; Li et al. 2018; Li et al. 2023; Qiang Zheng et al. 2016). Ouyang et al. described the development of a bioresorbable triboelectric sensor (BTS) for use in cardiovascular postoperative care (Fig. 3c) (Ouyang et al. 2021). Based on the triboelectric effect, BTS was designed to be implanted in the body and was capable of monitoring pressure changes in realtime. BTS has the potential to improve patient outcomes by providing accurate and continuous pressure monitoring without the risks associated with permanent implants. The human urinary system is regulated by the coordinated actions of the nervous and muscular systems. Any abnormalities in these systems may result in urinary disorders that can significantly affect daily life. Figure 3d, introduced a control system for monitoring bladder pressure and controlling urination (Arab Hassani et al. 2018). In this system, a TENG-based pressure sensor was employed to detect the filling state of the bladder. A bistable micro-actuator based on shape memory alloy was used to induce contraction and relaxation of the bladder for urination. This is a good try and provides a reliable approach for future clinical applications. Overall, these studies demonstrate the potential of self-powered, implantable sensors to revolutionize the field of biomedical monitoring. These sensors can enable accurate and continuous monitoring of various parameters without the need for frequent replacements or external power sources. As the technology continues to evolve, self-powered implantable sensors are expected to become an increasingly important tool in healthcare, particularly in the fields of cardiology, neurology, and postoperative care. Despite significant progress has been made, there is still room for improvement in the areas of novel materials, multimodal integration, and more effective tissue-device interfacing (Wen et al. 2023). For instance, the development of adhesive hydrogels that exhibit tissue adhesion properties can seamlessly secure implants onto biological tissue surfaces, enhancing the interface interaction while avoiding the damage caused by surgical sutures to the tissue surface (Wang et al. 2023). Considering the human body as a complex system, the monitoring of its health status necessitates the coordinated operation of multiple functional devices, imposing new requirements for system integration and information processing.

### Neural interfaces

**Fig. 4:**
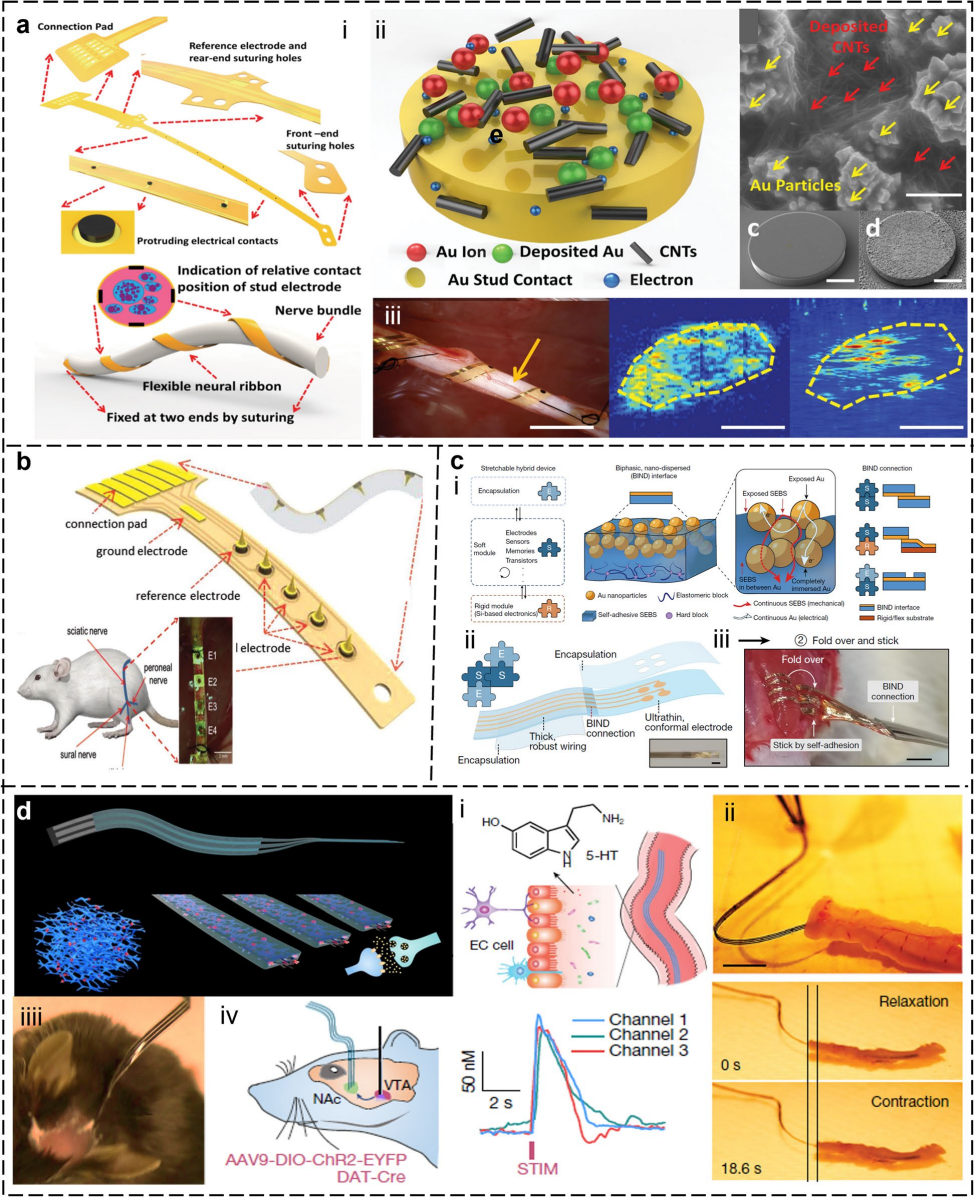
Neural signal sensing based on flexible neural interfaces. **a** The adaptive, no-invasive general neural ribbon electrode for small peripheral nerve signals recording. i: the neural ribbon electrode (NRE); ii: 3D contact point of nerve electrode; iii: in vivo performance of NRE (Xiang et al. 2016b). **b** A flexible nerve electrode with a 3D spike structure enables accurate monitoring and decoding of peripheral nerve signals for restoring motion perception (Wang, J. et al. 2018b). **c** A universal interface based on biphasic dispersed gold nanoparticles that can connect flexible/rigid modules in a plug-and-play way. i - ii: A schematic shows the integration way of flexible and rigid modulus; iii: Applying BIND electrode onto the peroneus longus muscle as an electrode (Jiang et al. 2023). **d** A soft and stretchable graphene-based biosensing neural interface for the brain and gut. i - ii: NeuroString for chemical sensing in the gut; iii - iv: NeuroString for brain neural signals recording (Li et al. 2022)

The nervous system is the human body's control center, regulating organ activity and mediating responses to external stimuli. Monitoring neural electrical signals or neurotransmitters provides insights into physiological and pathological processes, informing the prevention and treatment of neurological disorders (Cho et al. 2020; Lee, S. et al. 2017; Lee et al. 2022). However, it is not easy to accurately acquire and interpret the nerve signals, due to their features of weak strength and high noise (Cho et al. 2020; Xiang et al. 2016a). Furtherly, the conformal matching between the nerve electrode and the nerve tissue is also a crucial factor affecting the stability and biocompatibility of the neural interfaces (NIs) (Liu, Y. et al., 2020; Song et al. 2020). Various NIs such as electrode arrays, FETs, micro/nanomechanical systems (MEMS), and nanochannel arrays are utilized for measuring neural signals (Lee, Sanghoon et al., 2017). Meanwhile, microelectrode arrays, nanochannel arrays, and optical biosensors are commonly used for detecting neurotransmitter concentrations (Song et al. 2020; Wang, J. et al., 2018). Improving the adaptability and functions-long-term stability between NIs and neural tissue; improving the high spatial resolution of neural electrodes and developing neural electrodes that can be integrated with other neural technologies have been long-standing research priorities in this field. Figure 4a introduced a flexible and biocompatible neural ribbon electrode (NRE) that achieved self-adaptation to various diameter nerves by wrapping around nerve fibers (Xiang et al. 2016b). The NRE was coated with electrical-stable carbon nanotubes, which ensured a close 3D non-invasive contact with neural tissue and stable communication. In vivo experiments on rats showed that the NRE recorded neural signals from small nerves, such as the peroneal, tibial, and gastrocnemius nerves in a non-invasive way, which traditional cuff electrodes cannot achieve. With these unique functions, the neural ribbon electrode has the potential to specifically control target organs by communicating only with those closely connected to fine nerves and will help establish highly sensitive, real-time, and precise feedback to understand unknown electrochemical mechanisms. Compared to the surface potential signals, the internal signals of nerve fiber bundles contain more conduction and response related to the recovery of limb movement and sensory function. Figure 4b displayed a novel spiked ultra-flexible neural interface (SUNI), which collected sensory information from rat mechanoreceptors through the spike structure (Wang, J. et al., 2018). The novel 3D structural design of SUNI enables well-conformal contact with nerve fiber bundles. High-quality recordings from SUNI contributed differentiate tactile and proprioceptive stimuli, and furtherly provided for high spatial resolution classification of neural signals.

The mismatch between hard and hydrophobic NIs and the soft and wet nervous tissue is the most important cause of immune rejection and nerve electrode failure. To address this concern, the research hotspots mainly focus on three aspects. First, designing intrinsically hard micro-silicon-based devices with a flexible link structure, such as island-bridge structure, S-shape structure, wave structure, etc., endows implants with the ability to bend and stretch in a small range (Song et al. 2020). Secondly, using flexible materials as a bridge between implants and biological tissues (Liu, Y. et al. 2020b). Zhao et al. reported several examples to design flexible and adhesive hydrogels as surgical instrument coatings (Yu et al. 2019). Thirdly, developing intrinsically stretchable implants based on advanced soft materials. Figure 4c presented an ingenious method for the combination of a one-stop universal interface (UI) between rigid and soft modules (Jiang et al. 2023). This UI presented reliably connected soft, rigid, and encapsulation modules to form robust and highly stretchable electronics in a plug-and-play manner. The UI was composed of interpenetrating polymer and metal nanostructures that connected modules through simple pressing without the use of adhesives. The connection between soft-soft, and soft-hard modules both exhibited good mechanical and electrical stretchability. Additionally, electronics assembled with this interface were used for in vivo neural modulation and skin electromyography monitoring with high signal quality and mechanical robustness. These plug-and-play interfaces simplify and accelerate the development of skin and implantable stretchable electronics. For soft and stretchable implants, Liu et al. reported a morphing implantable electronic (MIE), which adapted to nerve tissue growth with minimal mechanical constraint (Liu, Y. et al. 2020b). The MIE consisted of a stretchable strain sensor and nerve electrode with stretchable poly (dimethyl siloxane)-isophorone bis urea (PDMS-IU) as the encapsulation layer and conductive polymer PEDOT: PSS as the conductive layer, which was able to monitor the growth of tissues and provide electrical stimulation at the same time. During the fastest growth period in rats, morphing electronics caused minimal damage to the rat nerve, which grows 2.4-fold in diameter. The MIE demonstrated the unique advantages of flexible and stretchable electronics for neural interfaces. Figure 4d gave another example from the same research group (Li et al. 2022). The researchers demonstrated a tissue-like stretchable sensor, named NeutroString to monitor the monoamine neurotransmitters. The NeuroString achieved electrochemical detection of different neurotransmitters by loading Fe_3_O_4_ or NiO nanoparticles onto graphene formed by laser-induced PEI, which addresses the weak strain limitation of monolayer graphene. At the same time, biocompatible polystyrene-block-pol y(ethylene-ran-butylene)-block-polystyrene (SEBS) was employed as the flexible substrate and encapsulation layer to realize tissue-like flexibility and mechanical modulus. The NeutroString enables a seamless interface with brain and gut tissue to realize chronic monitoring of neurotransmitters without disturbing the normal behaviors of experimental animals.

In summary, implantable neural interfaces have made significant progress in recent years, enabling new opportunities for understanding and interfacing with the nervous system (Cho et al. 2020; Lee, Sanghoon et al. 2017; Xiang et al. 2016a). These neural interfaces were designed to interact with the neural tissue, allowing for recording and stimulation of neural activity, and hold promise for treating neurological disorders and restoring lost functions. Even though, there are still significant challenges that need to be addressed for use as a clinical tool. One of the main challenges is the development of long-term stable interfaces that can withstand the harsh biological environment and avoid damage to the surrounding tissue (Song et al. 2020). Another challenge is the need for improved resolution and specificity of neural recording and stimulation, as well as the ability to interface with large populations of neurons (Li et al. 2022). Furthermore, the development of safe and effective clinical protocols for the implantation and monitoring of these devices, as well as regulatory and ethical considerations, are important areas for future research and development. Overall, the progress in implantable neural interfaces is promising, and continued research and development in this field will enable new opportunities for understanding and treating neurological disorders.

#### Flexible electronics for therapy

Recent advancements in wearable technology have revolutionized healthcare by enabling innovative therapeutic approaches that were previously impossible. With the introduction of flexible electronics, the development of intelligent wearable medical devices (IWMDs) has become a reality, facilitating real-time monitoring, diagnosis, and treatment of various medical conditions (Tan et al. 2022). These devices have gained immense importance in the quest for personalized healthcare and overcoming the limitations of passive therapy. Through tracking physiological and pathological factors and delivering therapeutic agents acting in an on-demand manner, IWMDs have become indispensable in providing predictive bio-analysis, timely treatment intervention, and improved drug efficacy, thereby promoting precision medicine (cheol Jeong et al. 2018; Tan et al. 2022).

**Fig. 5:**
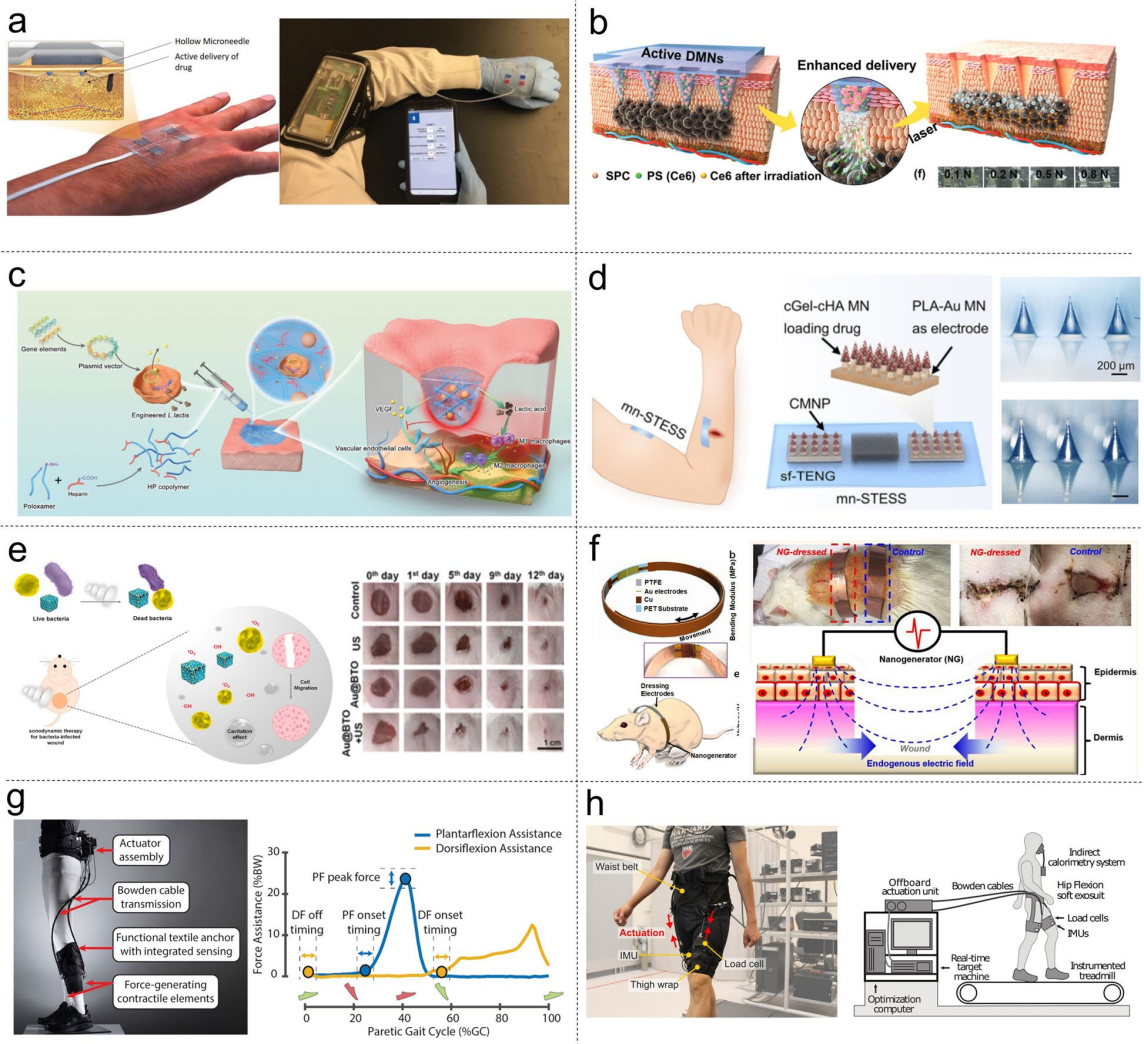
Wearable electronics for wound healing, drug delivery, and rehabilitation. **a** A wearable and programmable bandage that is able to transport essential pharmaceutical compounds and growth factors to the deeper regions of the wound site (Derakhshandeh et al. 2020). **b** A microneedle patch incorporating PS and O_2_ propellant, enabling more efficient transdermal delivery of PS and enhancing the effectiveness of photodynamic therapy. (Liu, P. et al. 2022b). **c** Engineering bacteria-activated thermos-responsive hydrogel to facilitate the healing of diabetic wound (Lu et al. 2021). **d** A self-sustaining transcutaneous electrical stimulation system utilizing microneedles to enhance the pharmacodynamics of epidermal growth factor (Yang, Yuan et al. 2022d). **e** A novel sonosensitizer, Au@BTO, incorporating Au nanoparticles into piezoelectric nanocomposite (BTO) with a modified Schottky junction for highly efficient sonodynamic therapy (Wu et al. 2021). **f** An electrical bandage to facilitate skin wound healing through electrical stimulation (Long et al. 2018). **g** An assistive and rehabilitative exosuit utilizing soft robotics for individuals recovering from stroke-related walking impairments. (Awad et al. 2020). **h** A lightweight and soft exosuit with optimized hip flexion assistance to reduce the energy expenditure of walking (Kim et al. 2022)

Wearable delivery devices for transdermal/topical drug delivery have gained popularity over traditional methods such as oral and injection delivery due to their flexibility and scalability. These devices have been used to deliver a range of therapeutic agents, including polypeptides, polysaccharides, small molecules, and growth factors, continuously and responsively (Kar et al. 2022; Yang, Yuan et al. 2022a; Yang, Yuan et al. 2022b). For instance, A. Tamayol’s group developed a wearable and programmable bandage, which incorporates polymeric miniaturized needle arrays (MNAs) to facilitate the targeted delivery of essential pharmacological agents and growth factors into the deeper layers of a wound (Fig. 5a) (Derakhshandeh et al. 2020). The bandage possesses the unique capability of actively delivering a diverse range of drugs with independent temporal profiles through the MNAs. The utilization of vascular endothelial growth factor (VEGF) as a therapeutic agent highlights the significance of not only choosing appropriate treatments but also considering the delivery technique and their spatial distribution within the wound bed. In the case of administering VEGF to chronic dermal wounds in diabetic mice using the programmable platform, notable enhancements are observed in wound closure, re-epithelialization, angiogenesis, and hair growth compared to the conventional method of topically applying therapeutics. Figure 5b presents a novel approach for achieving transdermal delivery of photosensitizers in a deeper and faster manner and improving photodynamic therapy (PDT) through the utilization of microneedle (MN) patches loaded with PS and O_2_ propellant (Liu, P. et al. 2022b). To enhance the PDT performance, sodium percarbonate (SPC) was incorporated into dissolving poly (vinyl pyrrolidone) microneedles. The inclusion of SPC in the microneedles resulted in a unique mechanism where it reacted with the surrounding fluid, generating gaseous oxygen bubbles. These bubbles induced vigorous fluid flows, leading to a significant enhancement in the penetration of chlorin e6 (Ce6) in hydrogel models and skin tissues. To evaluate the effectiveness of this gaseous oxygen-driven delivery of PS, a 20-day treatment was conducted on a tumor-bearing mice model. The results demonstrated a lower growth rate and reduced mass of the tumor, confirming the improved efficacy of PDT when using the proposed approach.

Figure 5c displays a delivery system consisting of living Lactococcus incorporated within a heparin-poloxamer thermos-responsive hydrogel. It aims to engineer the wound microenvironment and promote angiogenesis in a dynamic and temporal manner. By utilizing the living system, the production and protection of vascular endothelial growth factor (VEGF) is achieved, leading to enhanced proliferation, migration, and tube formation in endothelial cells. Additionally, the system secretes lactic acid, which helps shift macrophages into an anti-inflammatory phenotype, facilitating angiogenesis in diabetic wounds. Importantly, the delivery system can largely reduce the risk of systemic toxicities due to its capability in confining the bacterial population to wounds. Consequently , this proposed system demonstrates potential for therapeutic delivery with good efficiency, promoting rapid healing of wound microenvironment and holding promise as a scaffold in regenerative medicine applications (Lu et al. 2021). Recently, Yang et al. developed a microneedle-based self-powered transcutaneous electrical stimulation system (mn-STESS), which utilizes microneedles integrated with a sliding TENG to enhance the pharmacodynamics of epidermal growth factor, as shown in Fig. 5d (Yang, Yuan et al. 2022d). The primary objective of the mn-STESS is to improve the penetration and utilization of drugs by piercing the stratum corneum via microneedles. Additionally, it harnesses the mechanical energy generated from finger sliding to generate electricity, enabling transcutaneous electrical stimulation via the microneedles. This electrical stimulation serves as a supplementary factor that counteracts the decline in epidermal growth factor caused by glutathione and enhances the expression of its receptors in keratinocyte cells, effectively addressing the issue of receptor desensitization.

Sonodynamic therapy is a highly precise and minimally invasive approach for treating cancer and eliminating pathogens, which demands highly effective and biocompatible sonosensitizer. In a recent study, Wu et al. introduced a new type of sonosensitizer for highly effective sonodynamic therapy, which is a piezoelectric nanocomposite called barium titanate (BaTiO_3_, BTO) nano-cubes with a modified Schottky junction using gold nanoparticles (Au@BTO), as shown in Fig. 5e (Wu et al. 2021). By utilizing ultrasound as an external mechanical wave, the piezoelectric effect of Au@BTO is triggered, leading to enhanced separation and movement of charge carriers at the interface of the piezoelectric material and metal. This process significantly boosts the generation of reactive oxygen species (ROS) through a redox reaction. The exceptional efficiency of ROS production exhibited by Au@BTO makes it highly effective in combating both Gram-negative and Gram-positive bacteria. Moreover, both in vitro and in vivo experiments demonstrate that the sonodynamic treatment also promotes the migration of fibroblasts, which contributes to the healing of dermal wounds in mice. It has been widely recognized that electrical stimulations have proven to be another effective method for promoting the healing process of skin wounds. Figure 5f depicts an electrical bandage designed to accelerate skin wound healing, which generates an alternating discrete electric field through a wearable TENG that converts biomechanical energy from body movements into electricity (Long et al. 2018). Rat studies have shown that this approach leads to swift closure of a full-thickness rectangular skin wound within 3 days, which is notably faster than the healing processes based on contractions. In vitro studies suggest that the electric field stimulates the migration, proliferation, and trans-differentiation of fibroblasts, leading to accelerated skin wound healing.

In addition to the use of bioelectronics for therapy, there are also robotic exoskeletons that can directly assist or enhance human activities by integrating sensing, control, and computer science (Yang et al. 2008). In recent years, there has been significant progress in the development of exoskeletons specifically designed for the lower extremities. These exoskeletons aim to apply torques to biological joints, helping individuals with impaired ambulation capabilities or enhancing the abilities of healthy individuals. (Ding et al. 2018; Huo et al. 2014; Lee, G. et al. 2017; Quinlivan et al. 2017). These exoskeleton systems are typically robotic mechanisms consisting of rigid links, joints, and actuators that are arranged parallel to the lower limb and offer training or rehabilitation of gait. Professor C. Wash and his team have dedicated their efforts to the advancement of exosuits specifically designed for gait rehabilitation and effectively showcased a series of prototypes capable of assisting individuals with impaired mobility or augmenting the capabilities of healthy individuals through the reduction of metabolic rates. An example of their recent exosuit prototype is illustrated in Fig. 5g, which integrates Bowden cables, functional textile anchors, actuators, and contractile elements that generate forces (Awad et al. 2020). By connecting the Bowden cables to the shoe insole, assistive forces were effectively transmitted to the ankle. The incorporation of plantarflexor and dorsiflexor assistance within the soft exosuit exhibited promising outcomes in terms of enhancing walking speed and distance for post-stroke patients. Furthermore, an appropriately designed exosuit holds the potential to reduce the energy expenditure associated with walking in healthy individuals. In Fig. 5h, a soft and lightweight exosuit was developed to investigate the physiological and biomechanical effects associated with the hip flexion support (Kim et al. 2022). By employing human-in-the-loop optimization, it was found the exosuit can significantly reduce the energy expenditure associated with walking, achieving a nearly 50% decrease in mechanical power compared to existing state-of-the-art exosuits. The findings indicate that a tethered exosuit specifically designed to assist hip flexion can effectively lower the metabolic rate during walking by approximately 15.2 ± 2.6% when compared to unassisted walking. This reduction is equivalent to a 14.8% decrease in metabolic rate compared to walking without the exosuit. To provide assistance for individuals with limb impairments, prosthetics are designed to replace missing or amputated body parts, allowing users to regain functional mobility and perform daily tasks. These devices are typically custom-made to closely match the user’s anatomy and can be controlled through various mechanisms, such as muscle signals or sensory feedback. With ongoing technological advancements, these assistive devices are anticipated to become more sophisticated, customizable, and seamlessly integrated with the human body, ultimately enabling individuals to overcome physical limitations and achieve greater independence (Valle et al. 2021; Jean Won Kwak et al. 2020; Zhuang et al. 2019).

## VOC detection 

### Optical VOC gas identification

**Fig. 6:**
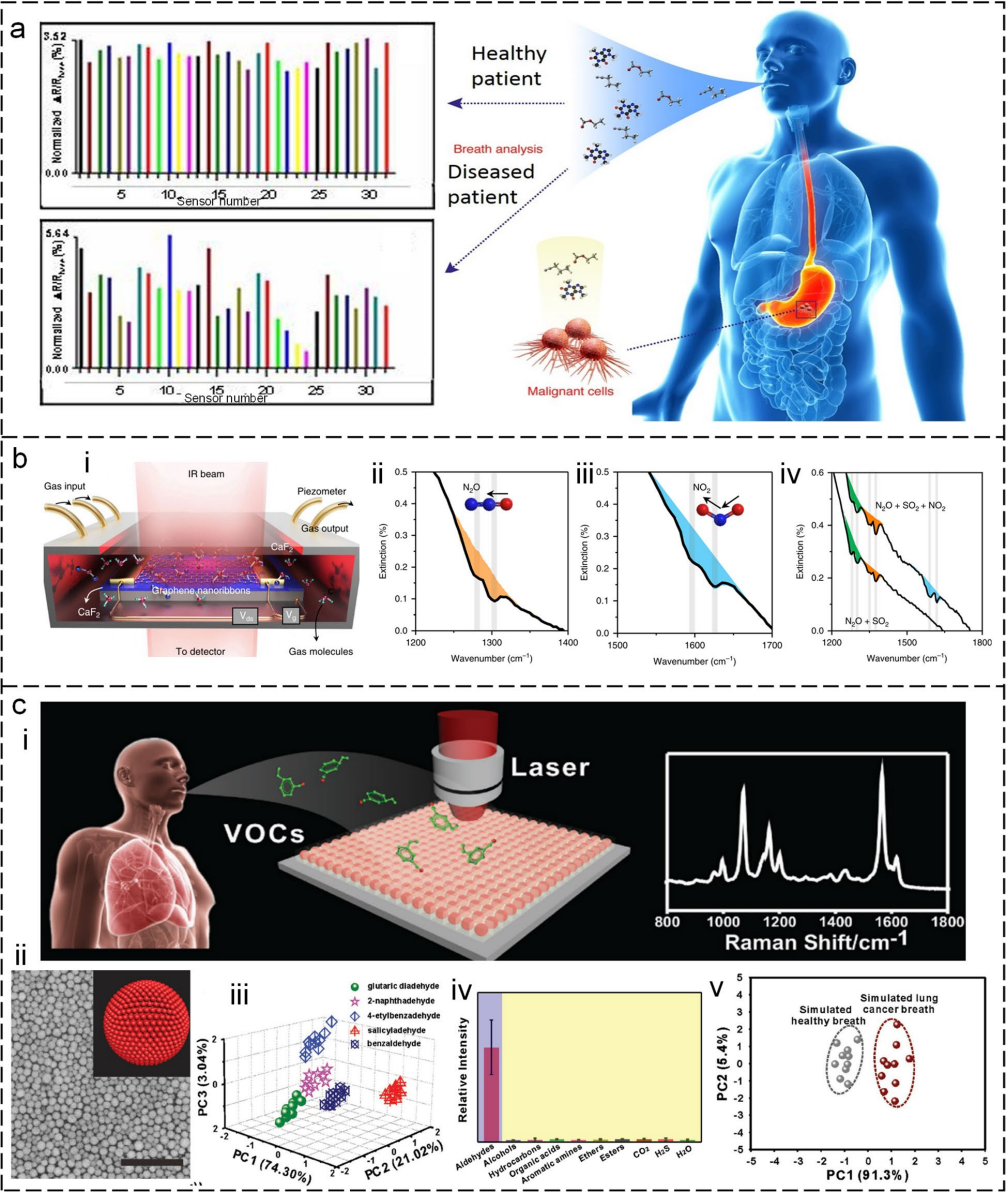
Optical volatile organic compound (VOC) gas identification for healthcare diagnoses. **a** VOC components in breath for disease diagnosis (Wilson 2018). **b** VOC gas identification using sensors based on the surface-enhanced infrared absorption (SEIRA) effect (Hu et al. 2019). i: Schematic representation. ii-iv: Identification of different nitrogen oxides. **c** VOC gas detection using sensors based on surface-enhanced Raman spectroscopy (SERS). i: Concept of the SERS platform. ii: SEM image of the SERS platform. iii: Demonstration of the detection of 5 VOC gases. iv: Selectivity of the SERS platform. v: PCA of a set of simulated healthy and lung cancer breath samples (Qiao et al. 2018)

Volatile organic compounds (VOCs) are gases that are released from various sources, such as chemicals, cleaning agents, building materials, and human bodies (Galstyan et al. 2021; Lim et al. 2021). The use of VOC gases in the breath as a diagnostic tool is a promising area of research. There is evidence to suggest that changes in the levels and types of VOC gases in breath can be indicative of various diseases and conditions, as shown in Fig. 6a (Binson and Subramoniam 2022; Wilson 2018). For instance, some studies have shown that VOC gases in breath can be used as biomarkers for the early detection of lung cancer (Hakim et al. 2012). Specific VOCs, such as benzene, toluene, and ethylbenzene, are elevated in the breath of lung cancer patients (Jalal et al. 2018). Changes in the levels of certain VOCs, such as ethane and pentane, have been associated with asthma (Peel et al. 2020). These VOCs are thought to be produced by oxidative stress in the airways and their levels have been shown to increase during asthma exacerbations. VOCs in breath can also be used as biomarkers for the detection and monitoring of diabetes (Behera et al. 2019). Changes in the levels of certain VOCs, such as acetone and isoprene, have been associated with diabetes and its complications.

One of the challenges in using VOC gases in breathing for disease diagnosis is the complexity and variability of the breath sample itself (Yan and Zhou 2022). The composition of exhaled breath can be affected by various factors, such as diet, age, smoking status, and medication use, which can make it difficult to identify disease-specific VOC signatures (Binson and Subramoniam 2022). In addition, the detection and measurement of VOCs in breath can be challenging due to the low concentration of many of these compounds, the presence of interfering compounds, and the need for sensitive and accurate detection methods. The development of ultrasensitive and selective VOC gas sensors is the key to facing these challenges. There are many methods available for the detection of VOCs, including electric and optical sensors (Khatib and Haick 2022). The key factors to evaluate a VOC gas sensor include 1) Sensitivity: which refers to its ability to detect low levels of VOCs in the air. High sensitivity is important for detecting trace amounts of VOCs that may be indicative of diseases (Wang, J.X. et al. 2020b); 2) Selectivity: Selectivity refers to the ability of a VOC gas sensor to distinguish between different types of VOCs. This is important because different VOCs can have different health effects and environmental impacts (Miah et al. 2022); 3) Response time: The response time of a VOC gas sensor refers to the time it takes for the sensor to detect changes in VOC concentration in the air. A fast response time is important for real-time monitoring of VOCs; 4) Stability and repeatability: The stability and repeatability of a VOC gas sensor refer to its ability to provide consistent and accurate readings over time (Liu and Zhang 2021).

VOC detection by infrared (IR) spectroscopy is advantageous (Lin and Xu 2020; Liu et al. 2021; Liu, X. et al. 2022d; Zhou, J. et al. 2023b; Zhu et al. 2021). Because it can not only detect the amount of VOC but also identify its type, which is very important for the diagnosis of diseases. However, the detection performance of this method is limited due to the low molecular absorption cross-section (10^-20^ cm^2^ per molecule in the mid-infrared). The infrared plasmonic nanoantenna is a type of artificially structured material that has been engineered to operate at the nanoscale level (Hui et al. 2021; Li, Dongxiao et al. 2021a; Xie et al. 2023; Xu, S. et al. 2022; Zhou et al. 2018; Zhou, H. et al. 2019a, 2019b). This technique is capable of manipulating light at extremely high levels through the process of exciting, localized surface plasmons, which creates an ultra-strong near-field (Xu, C. et al. 2022; Zhou, H. et al. 2020a; Zhou, H. et al. 2022a, 2022b). By trapping VOC molecules in the near-field of the nanoantenna, the IR absorption of these molecules can be amplified through a phenomenon known as surface-enhanced infrared absorption (SEIRA) (Liu, W. et al. 2022c; Ren et al. 2021; Xu et al. 2020). This is particularly useful since VOC molecules typically have a low IR absorption cross-section. A notable example of this approach was demonstrated by Hu *et al.* in 2019, who employed graphene nanoribbons to excite surface plasmons and detect the vibrations of multi-VOC gases, as shown in Fig. 6b (Hu et al. 2019). The mechanism of gas adsorption involved the adsorption and accumulation of gas molecules on the graphene layer through image attraction force and defect adsorption. The detection signal corresponded to a layer of gas molecules adsorbed on the graphene surface at a concentration of 800 zeptomoles per μm^2^. The study was successful in detecting several types of gas, including SO_2_, NO_2_, N_2_O, and NO, with a response time of 1 minute. Future research should focus on enhancing the crystal quality and mobility of graphene, which would improve the quality factor and field enhancement effect of graphene plasmons.

Surface-Enhanced Raman Scattering (SERS) is a potent analytical method that employs metallic nanoparticles to enhance the electromagnetic field, thereby amplifying the Raman scattering signal of VOC molecules (Lee et al. 2019; Wong et al. 2014). Gas-selective-capturing materials are critical in SERS-based VOC gas detection due to their benefits (Ly et al. 2021; Song et al. 2021). Firstly, these materials enhance the concentration of the target gas molecules, leading to an improved signal-to-noise ratio of the SERS measurement. The capturing materials allow the gas molecules to be concentrated in a small volume, resulting in a higher probability of interaction with the SERS-active metal nanoparticles. Additionally, the capturing materials facilitate a stable environment for the SERS measurement, preventing the target gas molecules from diffusing away from the detection region. Secondly, these materials improve the selectivity of the detection system by designing specific binding sites that can selectively recognize the target gas molecules based on their chemical properties, such as size, shape, and functional groups. The gas-selective-capturing materials can exist as porous materials, such as zeolites, metal-organic frameworks (MOFs), and porous polymers, or as surface-functionalized metal nanoparticles. For instance, Qiao and co-workers (Qiao et al. 2018) demonstrated a MOF-based SERS platform for VOC gas detection (Fig. 6c-i). To improve the adsorption of gaseous biomarkers for early detection of lung cancer, a ZIF-8 layer is applied onto the self-assembly of gold super-particles (GSPs) to reduce the flow rate of the gaseous biomarkers and to decrease the decay of the electromagnetic field around the GSP surfaces (Fig. 6c-ii). The gaseous aldehydes, which are indicators of lung cancer, are directed onto SERS-active GSPs substrates through a ZIF-8 channel. The gaseous aldehydes are then captured through a Schiff base reaction with 4-aminothiophenol pregrafted onto gold GSPs, resulting in a detection limit of 10 ppb. Combined with a machine learning algorithm, the platform can distinguish between lung cancer and healthy lung breath (Fig. 6c-iii, iv, v), which can be used as an in vitro diagnostic tool for early-stage lung cancer.

### Electronic VOC gas detection

**Fig. 7:**
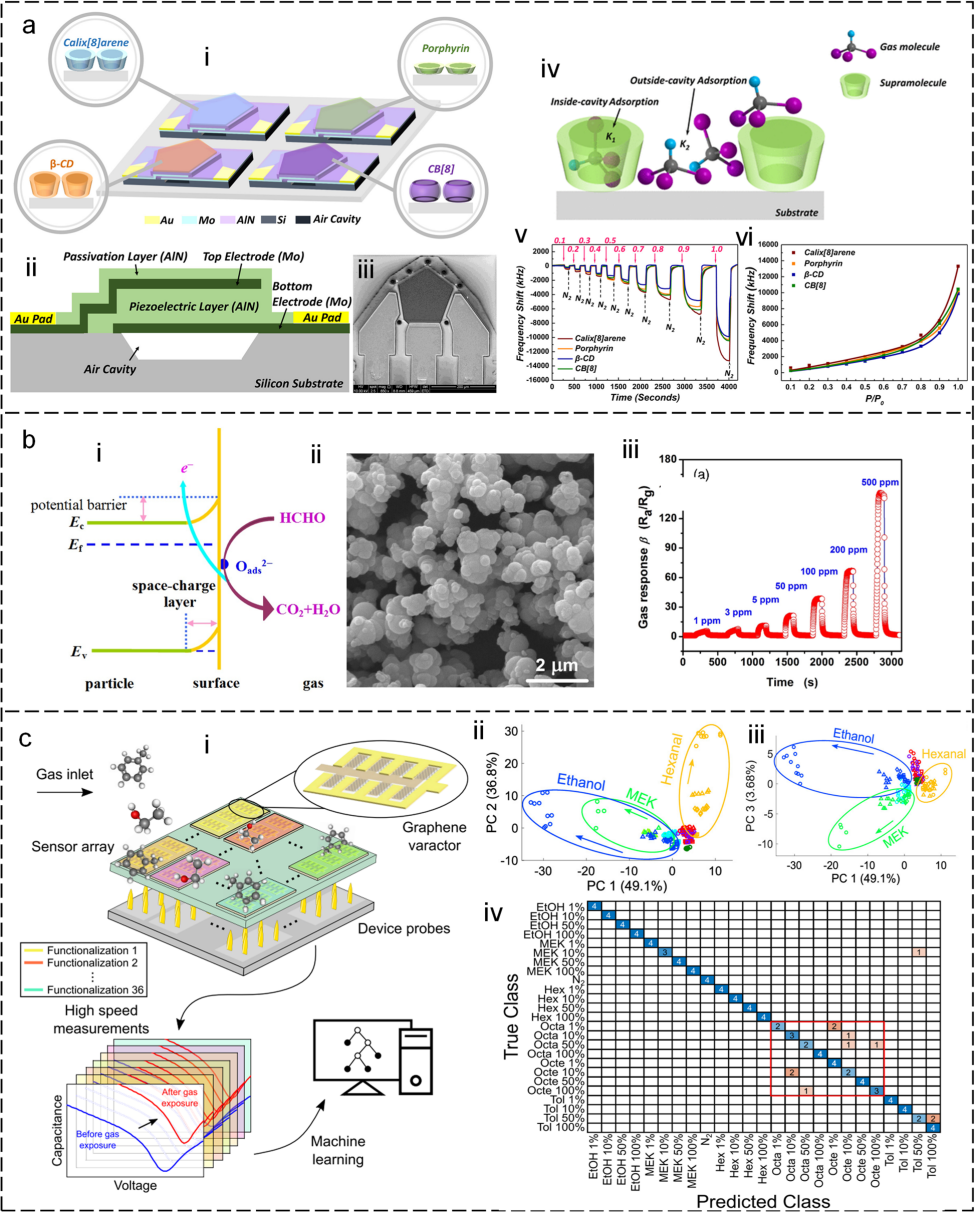
Electric methodology for VOC gas detection. **a** VOC gas detection using acoustic resonators based on the gravimetric mechanism (Lu et al. 2015). i: Schematic of the FBAR sensor array functionalized with four supramolecular monolayers. ii: Schematic of the device section. iii: SEM image of the device. iv: Schematic diagram of VOCs adsorption. v: Real-time responses of the FBAR sensor with exposure to chloroform gas. vi: Adsorption isotherms of chloroform. **b** VOC gas detection using semiconducting metal oxide (Li et al. 2017). i: A Reaction mechanism of the sensor for HCHO detection. ii: SEM images of as-synthesized SnO_2_ microspheres. iii: Dynamic response-recovery curve of the SnO_2_ microspheres gas sensor. **c** Machine learning-enabled graphene electronic nose for VOC gas detection (Capman et al. 2022). i: Schematic of the ML-enabled sensor system. ii: PCA score plots showing the projection of the measured responses onto principal components 1 and 2. iii: Confusion matrix

The technique of detecting VOCs using electrical signal output has gained significant interest due to the lack of necessity for light sources and detectors. Representative methods include acoustic wave devices (AWDs) based on the piezoelectric effect (Chen et al. 2018; Dou et al. 2018; Zhang et al. 2018), semiconductor metal oxide (Lin et al. 2019), and two-dimensional materials (Sun et al. 2018). The AWD typically consists of a piezoelectric substrate and a sensing film that is coated on the surface of the substrate (McGinn et al. 2020). When an electrical signal is applied to the piezoelectric substrate, it generates an acoustic wave that propagates through the substrate and the sensing film. The sensing film is designed to selectively interact with specific gas molecules, causing a mass-loading effect that alters the resonant frequency of the device. As VOCs interact with the sensing film, they are adsorbed onto the surface, causing a change in the mass of the film, and altering the resonant frequency of the device, referred as to the gravimetric sensing mechanism. This change in frequency is detected and measured by the device, providing information about the presence and concentration of the VOCs. For instance, Lu *et al.* (Lu et al. 2015) conducted a study where they developed four film bulk acoustic resonators (FBARs) with an ultrahigh operating frequency of 4.4 GHz to detect VOC gases (Fig. 7a-i, ii, iii). Each resonator was coated with a different supramolecular monolayer as the sensing film, namely p-tert-butyl calixarene (Calixarene), 2, 3, 7, 8,12, 13, 17, 18-octaethyl-21H, 23H-porphine (Porphyrin), β-cyclodextrin (β-CD), and cucurbituril (CB). The supramolecular monolayer coatings facilitated fast and reversible detection of VOCs by allowing the monitoring of the gas-phase “host-guest” interaction with the high-frequency FBAR sensor vibrating at 4.4 GHz (Fig. 7a-iv, v). This study highlights the potential of supramolecular coatings to enhance the performance of sensors for gas detection applications. Notably, gas selective adsorption films play a crucial role in gas sensors, offering several primary functions and benefits (McGinn et al. 2020). First and foremost, these films are designed to enhance selectivity. Through tailoring their composition, gas selective adsorption films exhibit a strong affinity for the specific gas species of interest, ensuring that only the desired gas molecules are adsorbed. This selectivity enhancement enables the sensor to accurately detect and quantify the target gas. Furthermore, gas-selective adsorption films contribute to sensitivity improvement. By preferentially adsorbing the target gas molecules onto their surface, these films amplify the sensor's sensitivity, allowing for the detection of even trace amounts of the gas. Additionally, these films aid in mitigating interference caused by other gases present in the surrounding environment. They are engineered to minimize or suppress the adsorption of interfering gases, thereby reducing their impact on the sensor's accuracy and specificity. Finally, gas selective adsorption films are characterized by their stability and repeatability in gas adsorption. Their engineered composition ensures long-term stability and reversibility, enabling consistent and reliable performance of the gas sensor over-time. Semiconducting metal oxides (SMOs) are widely used for VOC detection due to their high sensitivity and low cost (Steinhauer 2021). The basic mechanism of SMO sensors for VOC detection involves the change in the electrical conductivity of the sensor material in the presence of target VOC molecules. The sensor material is typically a metal oxide, such as tin dioxide (SnO_2_) (Fazio et al. 2021), tungsten oxide (WO_3_) (Modak et al. 2023), or zinc oxide (ZnO) (Pargoletti and Cappelletti 2020), that is highly sensitive to changes in the ambient environment (Ou et al. 2022; Pargoletti and Cappelletti 2020). When the sensor material is exposed to VOCs, the molecules are adsorbed onto the surface of the metal oxide, leading to a change in the electrical conductivity of the sensor material. This change in conductivity is due to the interaction between the adsorbed molecules and the free electrons in the metal oxide lattice. The presence of VOCs increases the number of free electrons, which in turn increases the conductivity of the sensor material. The change in conductivity can be measured by applying a small voltage across the sensor material and measuring the resulting current. The magnitude of the current is proportional to the concentration of the target VOC in the ambient environment. By monitoring the change in conductivity over time, the sensor can detect the presence of VOCs and provide information on their concentration. For instance, Li *et al.* (Li et al. 2017) prepared SnO_2_ microspheres for formaldehyde gas sensing by a simple hydrothermal method (Fig. 7b-i, ii). Upon exposure to the testing gas atmosphere, the sensor surface absorbed oxygen species which in turn captured free electrons from the sensing materials. This process resulted in the ionization of SnO_2_ microspheres to adsorbed oxygen ions at grain boundaries. The results show that the sensor has a response value of 38.3 to 100 ppm formaldehyde at an operating temperature of 200 °C, and the detection limit reaches 1 ppm (Fig. 7b-iii).

To enable accurate measurement of multiple gases, the combination of diverse gas-sensing films and an array design assisted by artificial intelligence algorithms shows great potential (Barucha et al. 2022; Yang et al. 2018). Capman *et al.* (Capman et al. 2022) demonstrated the selective and rapid detection of multiple VOCs using graphene-based variable capacitor arrays (Fig. 7c). The functionalization of graphene with different chemical acceptors enabled the development of arrays containing 108 sensors with 36 chemical receptors for cross-selectivity. Multiplexer data acquisition from 108 sensors was achieved in tens of seconds, allowing for rapid measurement of the VOCs. Despite the reduced signal amplitude associated with the rapid measurement, the method demonstrated a significant 98% accuracy in the detection of the five tested analytes (ethanol, hexanal, methyl ethyl ketone, toluene, and octane). The addition of 1-octene, an analyte with a structure highly like octane, resulted in a slightly reduced accuracy of 89%. These findings suggest the potential of graphene-based varactor arrays for the selective and rapid detection of multiple VOCs at different concentrations.

In addition, optical and electrical VOC detection methods have their own strengths. Optical methods for VOC gas detection offer high sensitivity, excellent selectivity, and fast response times (Khan et al. 2020). They are capable of detecting low gas concentrations in the parts per billion (ppb), making them suitable for applications requiring high sensitivity. The use of specific wavelengths of light allows for the discrimination of target gases from interfering gases, enhancing selectivity. Optical methods also provide real-time or near real-time monitoring capabilities, with response times typically in the order of seconds or milliseconds. However, they may be susceptible to interference from environmental factors and can be costlier due to specialized optical components. On the other hand, electrical methods exhibit good sensitivity in the parts per million (ppm) range and offer flexibility in sensor design (McGinn et al. 2020). They can be integrated into compact and portable devices, allowing for personal monitoring or handheld applications. While electrical methods may have slightly lower sensitivity compared to optical methods, they can compensate with the use of sensor arrays and pattern recognition techniques to enhance selectivity. Electrical methods generally have faster response times and can be more resilient to environmental interferences. However, they may require periodic calibration or replacement of sensing materials, and their selectivity may be limited for individual sensors. Ultimately, the choice between optical and electrical methods depends on specific application requirements, target gas characteristics, and cost considerations.

### AI-enhanced VOC platform

**Fig. 8:**
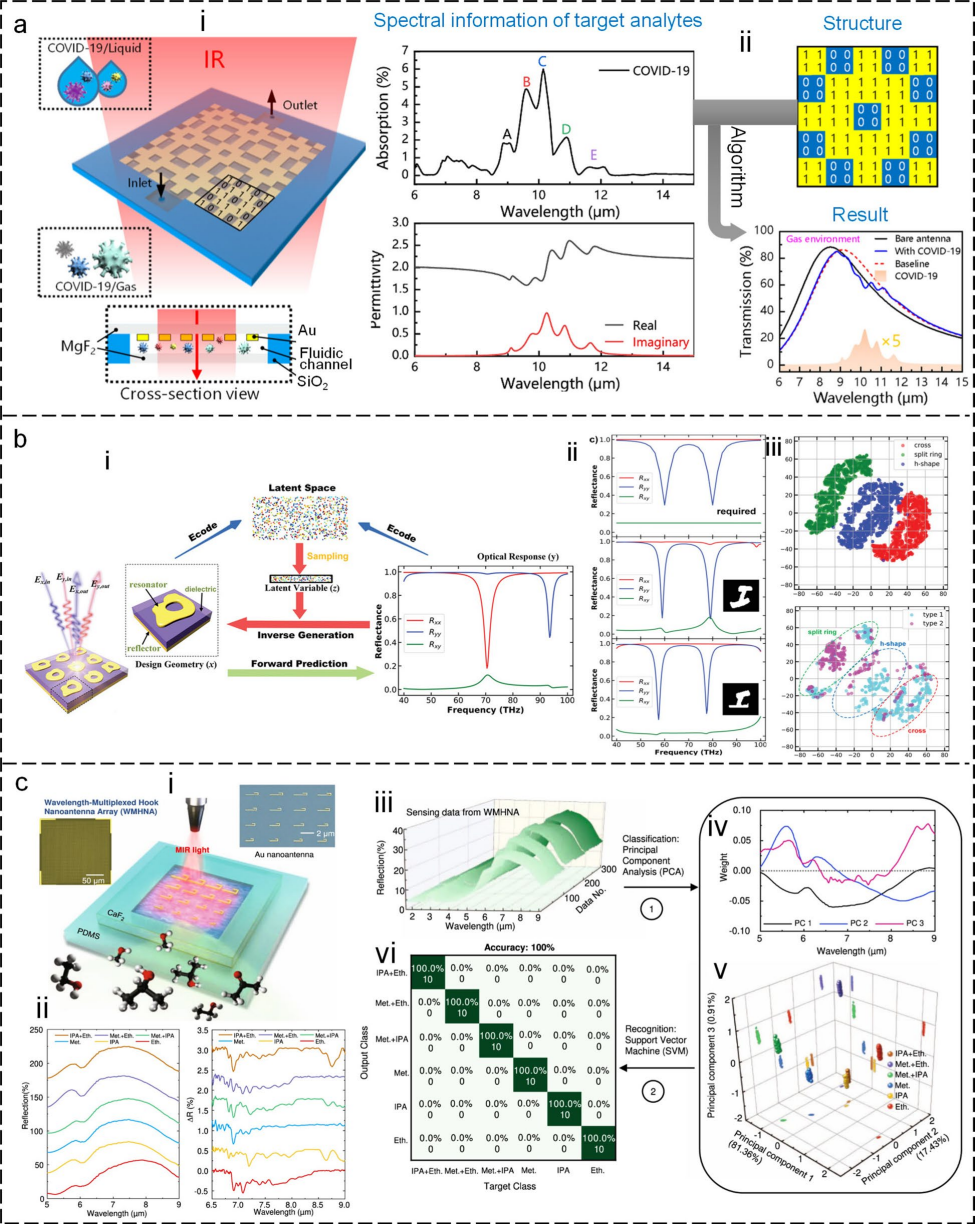
Artificial intelligence-enhanced sensor design and VOC detection. **a** Machine learning-enabled forward design of sensors (Li, D. et al. 2021b). i: 3D view of the sensor. ii: Design process. **b** Inverse design of sensors using a semi-supervised deep learning algorithm (Ma et al. 2019). i: A schematic of inverse design. ii: The desired spectra (upper panel), the results of inverse design (lower panel). Insets are the design pattern through algorithms. iii: Visualization of the latent space. **c** Machine learning-enabled VOC detection (Ren et al. 2022). i: A diagram illustrating the VOC sensor. ii: The measured spectra of analytes, show spectral overlapping, which is difficult to distinguish clearly with the classic data processing methods. iii: The measured raw spectral data. iv: Data preprocessing using PCA. v: The weight of scores of each spectrum in 3D space. vi: The confusion map for analyte classification results

AI algorithms are becoming increasingly important for VOC gas detection because of their benefits for VOC sensors (Haripriya et al. 2023; Lekha and Suchetha 2021; Lotsch et al. 2019; Mahmood et al. 2023; Thrift et al. 2019; Zhou et al. 2021; Zhou, H. et al. 2023a). First, the use of machine learning algorithms facilitates the automated design of volatile organic compound sensors, thereby eliminating the need for arduous and time-consuming design processes. This is exemplified in the design of SEIRA’s antenna, which involves the analysis of the analyte molecule’s infrared spectrum and the subsequent selection of an appropriate antenna structure to match molecular vibrational frequencies. The design process involves critical and repetitive optimization procedures, including the selection of structural shapes, tuning of antenna dimensions, and consideration of nanofabrication limitations. AI-assisted design systems efficiently handle these tasks, leading to more effective and efficient design outcomes. For instance, Li and co-workers (Li, D. et al. 2021a, 2021b) developed a sensitive SEIRA substrate for COVID-19 detection using a genetic algorithm (GA)-based AI approach in 2021 (Fig. 8a-i). The currently available COVID-19 diagnostic methods, such as RT-PCR, serological tests, or chest computed tomography scans, have their limitations, such as low sensitivity and time-consuming procedures. The development of plasmonic methods for point-of-care diagnostics is highly desirable. The GA-based AI approach employed molecular complex permittivity extraction from the infrared spectrum as the first step for modeling the vibrational behavior of molecules. Subsequently, the GA rapidly identified the optimal solution with high sensitivity, zero detuning of plasmonic resonance and molecular vibration, and high enhancement factor from multi-design parameter problems (Fig. 8a-ii). Additionally, SEIRA methods can differentiate the mutation of COVID-19, which presents a significant challenge to conventional diagnostic methods, by comparing the intensity and frequency of virus peaks.

In addition to the forward design described above, the AI algorithm can also be used in the reverse design, resulting in a reduced amount of training data through the utilization of both labeled and unlabeled data (Liu et al. 2018). Ma *et al.* (Ma et al. 2019) present a unique network for an inverse design that utilizes predefined distribution-based latent variables to encode the structural design and optical responses from input geometry (Fig. 8b-i). The latent variables can be randomly sampled and decoded to reconstruct the original structural geometry, thus enabling inverse design. The simulated spectra obtained from the inverse design parameters (middle and bottom pane in Fig. 8b-ii) closely match the required spectrum (upper panel). The sampling process produces various outputs for the same target spectrum, generating multiple candidates for reverse design. Moreover, the proposed model can learn to differentiate between various shapes via encoding-decoding training iterations on both labeled and unlabeled data (Fig. 8b-iii).

AI algorithms can facilitate the analysis and processing of data by VOC sensors in a prompt, direct, and automated manner (John-Herpin et al. 2021; Kuhner et al. 2019). This relieves the pressure caused by massive spectral data through dimensionality reduction. In real-time monitoring applications, the output data consists of three-dimensional information, encompassing spectral intensity, wavelength, and time. If multiple analytes are targeted, the information becomes four-dimensional, with category information included. This leads to a considerable volume of spectral data that optical methods struggle to analyze and process accurately and quickly. PCR, among other ML algorithms, can reduce the dimensionality of information while preserving features. This leads to a decrease in the amount of data, simplification of data processing, and prompt generation of test results. As technological advancements in sensors and the number of gases continue to increase, it is foreseeable that data volume will inevitably rise. Therefore, dimensionality reduction of spectral data using ML algorithms is an invaluable aid to VOC sensors. Ren *et al.* (Ren et al. 2022) designed a hook-shaped nanoantenna array that utilizes wavelength multiplexing for continuous broadband detection of multiple absorption peaks in the fingerprint region (Fig. 8c-i). The SEIRA spectra of different analytes with similar functional groups tend to overlap in many regions, resulting in difficulty distinguishing them in mixtures with narrow-band SEIRA substrates (Fig. 8c-ii). However, through the integration of PCA and SVM algorithms, the authors have achieved 100% recognition accuracy for the identification of methanol, ethanol, and isopropanol, as demonstrated in Fig. 8c-iii-vi. Proper preprocessing techniques are employed to convert raw sensor data into a format that is more appropriate and informative for the learning algorithm (Guo, K. et al. 2021a). The preprocessing of sensor data involves several key steps. Firstly, data cleaning is performed to address missing data, outliers, and noise in the sensor readings. Missing data can be handled through techniques such as mean imputation, interpolation, or specialized machine-learning algorithms designed for handling missing data. Secondly, data normalization or scaling techniques, such as min-max scaling or z-score normalization, are applied to address variations in scales and ranges observed in sensor data, as these can impact the performance of machine learning models. By bringing all features to a similar scale, these techniques ensure that no single feature dominates the learning process. Additionally, feature selection or extraction methods are employed to identify the most relevant features from the potentially large number of features present in sensor data. Techniques such as correlation analysis, mutual information, or feature importance measures are commonly used for this purpose. Finally, the preprocessed sensor data is divided into training, validation, and testing sets. The training set is utilized for training the machine learning model, the validation set assists in tuning hyperparameters, and the testing set is employed to evaluate the final performance of the trained model. Overall, the advances in artificial intelligence techniques offer the promising potential to enhance VOC sensors through rapid sensor design and automated data processing, thus enabling them to effectively address the challenges of the future. Besides, physical knowledge can improve the algorithm’s performance and reduce the difficulty of optimization. For instance, Khatib et al. demonstrated that incorporating knowledge of the underlying physics as input and pre-training these quantities during the training process holds the potential to enhance network performance and mitigate the challenges associated with optimization (Khatib et al. 2022). The model's ability to accurately predict the spectrum of interest, as evidenced by its agreement with the target spectrum, serves as a testament to the efficacy of deep neural networks in optimizing the design of VOC gas sensors. Furthermore, the Mean Squared Error computed for the cross-validation set post-training serves as a valuable metric for assessing the prediction accuracy of the model.

## Human-machine interfaces

The rapid development of sensor technology has been a driving force behind Industry 4.0 (Wang, T. et al.2018c; Zhou et al. 2015; Zhu et al. 2019). Sensors that can detect and respond to physical stimuli or chemical variations have enabled various systems, like cameras for visual sensing and microphones for voice recognition, to collect and process data in real-time. MEMS-based sensors, such as accelerometers, gyroscopes, pressure sensors, tactile sensors, and biosensors, have found extensive use in numerous applications, particularly in wearable electronics, due to their numerous advantages, including small size, low power consumption, low cost, high reliability, and robustness (Erdil et al. 2022; Liu et al. 2013; Manvi and Mruthyunjaya Swamy 2022; Pitchappa et al. 2015; Yazici et al. 2020). The integration of AI data analytics with wearable sensors allows for the capture of crucial information, such as muscle deformation, joint bending, temperature changes, and heartbeat frequency. This data is invaluable for a wide range of applications, encompassing healthcare, environmental monitoring, human-machine interactions (HMI), and smart city. By harnessing advanced sensor technology and AI data analytics, wearable devices can offer real-time insights, facilitating smarter decision-making and enhancing efficiency across various applications (Han et al. 2018; Liu, H. et al 2020b; Wang, Y. et al., 2021).

### Tactile sensor

**Fig. 9:**
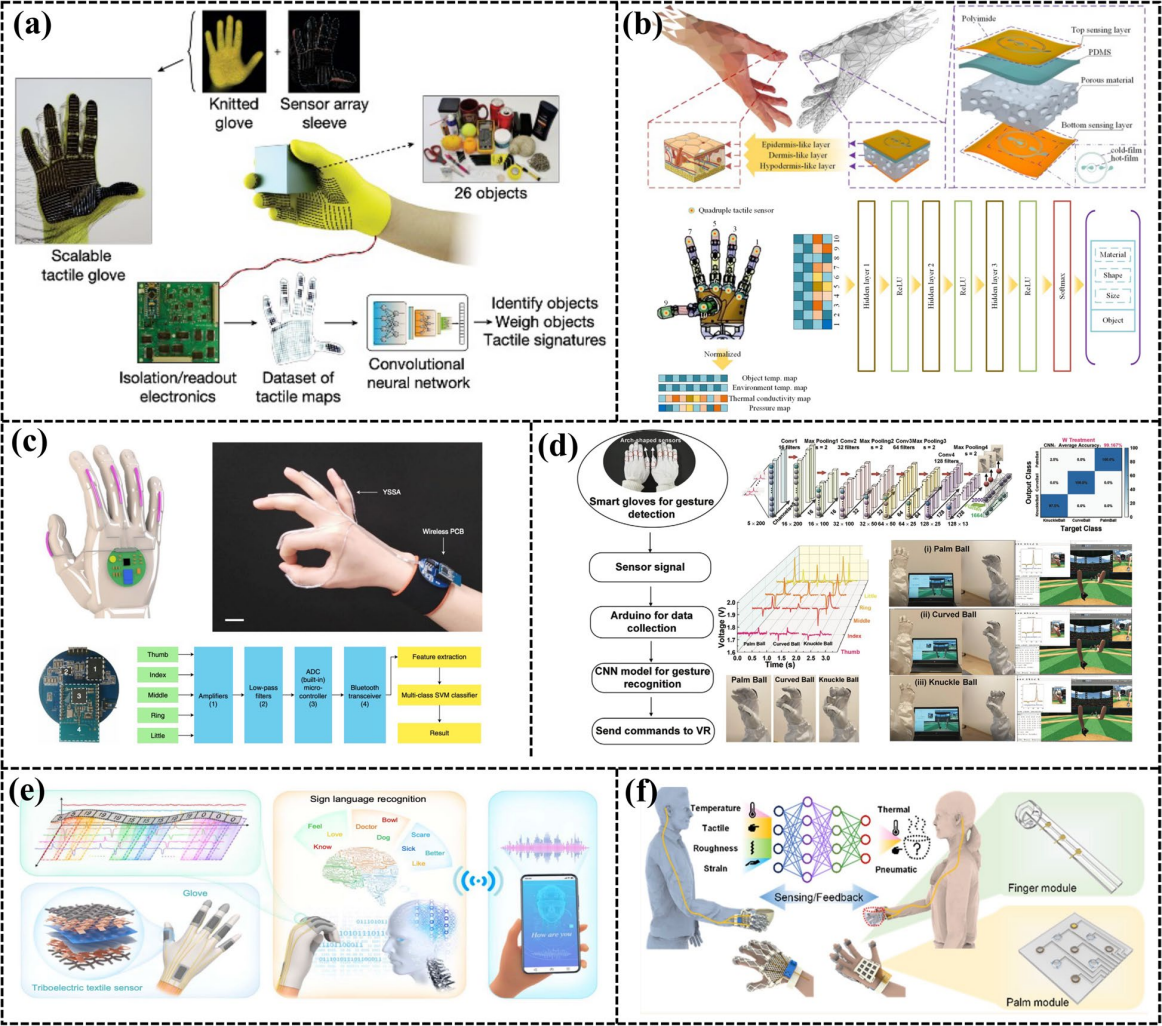
Advanced AI-enhanced wearable glove sensors. **a** A scalable tactile glove (STAG) consists of a sensing sleeve with 584 piezoresistive sensors (Sundaram et al. 2019). **b** A flexible quadruple tactile sensor for a robot hand to perceive grasped objects (Li et al, 2020). **c** A TENG strain sensor based on a unique yarn structure for the smart glove application (Zhou, Z. et al. 2020b). **d** A low-cost, self-powered, and intuitive glove-based HMI combining superhydrophobic triboelectric textile sensors (Wen, F. et al. 2020a, 2020b, 2020c). **e** A sign language recognition and communication system based on TENG gloves (Wen, F. et al. 2021)

The subsequent examples aim to emphasize the significance of incorporating tactile sensors. As illustrated in Fig. 9a (Sundaram et al. 2019), Sundaram et al. propose an affordable and scalable tactile glove (STAG) capable of object identification, weight estimation, and hand pose recognition. The STAG comprises a sensing sleeve with 584 piezoresistive sensors, a knit glove, and readout electronics that utilize the ResNet-18 architecture to train the model and ultimately recognize 26 distinct objects. These methods demonstrate that a substantially larger amount of information becomes available for examining interactions at a more profound level, thanks to the advancements in flexible electronic components and the support of DL techniques, thus facilitating the future design and development of next-generation wearable electronics and systems.

As portrayed in Fig. 9b, Li et al. develop a flexible quadruple tactile sensor that enables a robotic hand to perceive grasped objects of varying materials and shapes and further employs a multilayer perceptron (MLP) with three hidden layers to achieve automatic waste classification. The design of the tactile sensor includes two sensing layers, each with two sensing elements, enveloping a porous silver nanoparticle-infused polydimethylsiloxane (PDMS). The top and bottom hot films respond to the thermal conductivity of the object touched and the pressure exerted respectively, influenced by the varying thermal conductivity of diverse materials and the change in thermal conductivity of porous substances caused by deformation. The cold films serve as local temperature sensors, identifying objects and environmental temperatures. The tactile sensors can detect multiple stimuli simultaneously with negligible cross-coupling errors, offering more insightful features related to the objects and improving recognition precision during the ML process. Hence, it proves its potential in substantially lessening the load on individuals for environmental protection and sustainable progress in smart homes.

### AI-enhanced HMIs

In comparison to conventional and prevalent resistive and capacitive sensors, piezoelectric sensors and triboelectric sensors can generate self-produced voltage upon mechanical deformation, removing the need for external power sources. Zhou et al. propose a TENG strain sensor based on a distinctive yarn structure, as depicted in Fig. 9c (Zhou, Z. et al. 2020b). The core of the sensor unit comprises a conductive yarn wrapped around a rubber microfiber, all of which are housed within a PDMS sleeve. Various degrees of deformation induce a steady and ongoing change in the contact surface between the PDMS sleeve and the coiled conductive yarn, granting the sensor remarkable linearity and sensitivity across a wide strain range (20–90%). Once a wireless printed circuit board (PCB) is integrated for signal collection, processing, and transmission, a wearable sign-to-speech conversion system can be developed using the multi-class SVM algorithm. The system maintains an overall precision above 98.63% and exhibits a quick response time (less than 1 second). This shows an economical approach to enable communication between individuals who use sign language and those who do not, and it also highlights the potential use of TENG-based HMI in healthcare settings.

In addition, a low-cost, self-powered, and intuitive glove-based HMI is developed by combining superhydrophobic triboelectric textile sensors with ML, as shown in Fig. 9d (Wen, F. et al. 2020a, 2020b, 2020c). This innovative design allows for complex gesture recognition and control in both real and virtual spaces while minimizing the negative effects of humidity and sweat on performance. A carbon nanotubes/thermoplastic elastomer (CNTs/TPE) coating method is used to create superhydrophobic textiles, resulting in improved energy harvesting and human motion sensing capabilities. This textile has a quicker recovery time from high-humidity environments, threefold boosted triboelectric performance, and better biomechanical energy scavenging compared to pristine textiles. The glove-based HMI, enhanced with machine learning, demonstrates a high recognition accuracy of 96.7%, outperforming non-superhydrophobic systems (92.1%). It also maintains 80% voltage output even after an hour of exercise. The developed glove interface has been successfully applied to various virtual reality/augmented reality (VR/AR) controls, including shooting games, baseball pitching, and flower arrangement.

Moreover, glove-based gesture recognition systems hold great potential for assisting the speech and hearing impaired, particularly in sign language recognition. AI-enhanced glove systems can effectively recognize and translate various sign language gestures in real time, facilitating seamless communication for the speech and hearing impaired. Additionally, these advanced systems can be further refined by training them on diverse and extensive datasets, improving their accuracy and recognition capabilities for a universal platform to recognize complex gestures in various applications. Therefore, as shown in Fig. 9e Wen et al. demonstrate a sign language recognition and communication system based on smart glove sensors (Wen, F. et al. 2021). The deep learning algorithm initially identifies word components, followed by the reconstruction of the original sentences, achieving respective accuracies of 82.81% and 85.58%. In addition, the segmentation method opens new avenues for recognizing novel or previously unseen sentences. Specifically, recognized word units can be reorganized into a different sequence to construct fresh sentences. Concurrently, the deep learning model identifies all fundamental word elements in the new sentence and suggests an appropriate translation. This way, sentences not included in the training dataset can be recognized. Lastly, sentence recognition results are visualized in a virtual space where sign language users can communicate using their familiar language, while non-signers can directly input via their controlled VR interface. This breakthrough in recognizing both existing and new sentences enhances the practicality of sign language recognition systems, marking a significant step towards minimizing communication obstacles between sign language users and non-signers.

## AI-enhanced multimode sensor

**Fig. 10:**
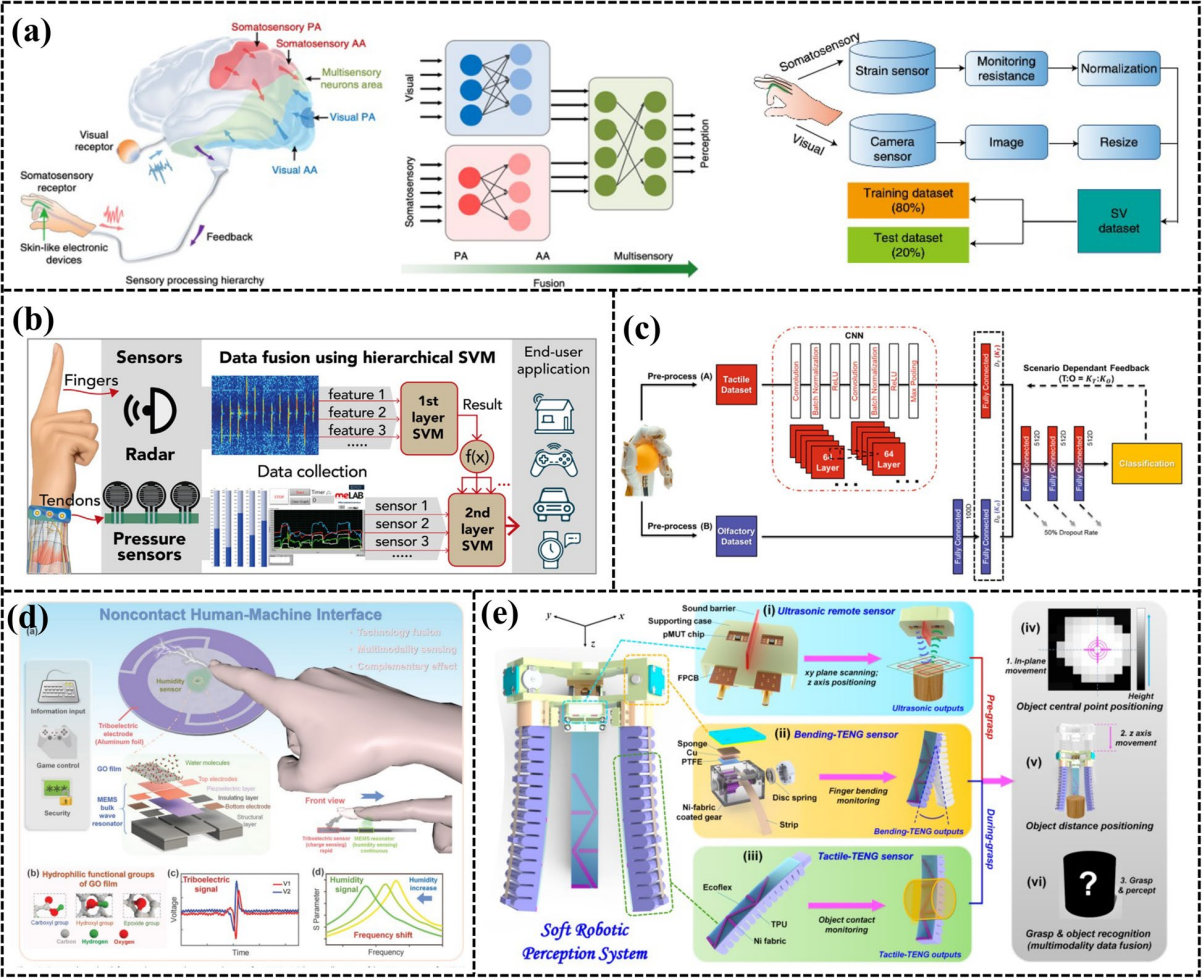
Multimodal sensors and ML-base data fusion. **a** Bioinspired data fusion architecture by integrating visual data with somatosensory data from skin-like stretchable strain sensors (Wang, M. et al. 2020a, 2020b, 2020c). **b** HSVM algorithm for radar and pressure sensors (Liang et al. 2019). **c** A mole-inspired olfactory-tactile-associated ML architecture (Liu, M. et al. 2022c). **d** A multimodal noncontact HMI by combining a MEMS humidity sensor and a triboelectric sensor (Le et al. 2022). **e** A soft robotic perception system that integrates an ultrasonic sensor with flexible triboelectric sensors (Shi et al. 2023)

Multimodality offers numerous benefits, including improved accuracy, redundancy, complementary information, adaptability, robustness, enhanced functionality, and scalability (Sun et al. 2021; Shih et al., 2020). These advantages make smart sensor systems an essential component of various fields, such as robotics, environmental monitoring, healthcare, and security, where reliable and comprehensive information is vital for effective decision-making and performance, allowing smart homes to make informed decisions and customized actions. As depicted in Fig. 10a (Wang, M. et al. 2020a, 2020b, 2020c), a bioinspired data fusion architecture was crafted to carry out human gesture recognition, combining visual data with somatosensory data from skin-like stretchable strain sensors. The strain sensors were fabricated from single-walled carbon nanotubes, and the learning architecture utilized a CNN for visual processing, succeeded by a sparse neural network for sensor data fusion and recognition at the feature level. This method of data fusion achieves a stellar recognition accuracy of 100%, while maintaining this accuracy under less than non-ideal conditions for the image sensor, such as noise, under-exposure, or over-exposure. The illustration of robot navigation via hand gestures showcases the stability of this data fusion method with an error margin of 1.7% under regular illumination and 3.3% in darkness.

Additionally, a novel method of data fusion from multiple sensors using a hierarchical SVM (HSVM) algorithm is presented in Fig. 10b (Liang et al. 2019). It employs an innovative learning system, merging radar technology to track the movements of the hand and fingers, along with a versatile array of pressure sensors, determining the pressure distribution surrounding the wrist. With the aim of efficiently amalgamating diverse data types, we designed the HSVM framework. It considers various factors including the sampling rate, data architecture, and intricate gesture data from the pressure sensors and radar. Upon analyzing the datasets collected from a wide range of 15 participants, it was observed that standalone radar technology resulted in a decent average classification accuracy of 76.7%, while the pressure sensors alone clocked an accuracy of 69.0%. However, a significant improvement was noticed when the HSVM algorithm was implemented, as it combines the outputs from both the pressure sensors and radar, pushing the classification accuracy rate to a commendable 92.5%.

Furthermore, a bioinspired olfactory-tactile (BOT) associated machine-learning architecture was proposed to achieve object recognition by processing multimodal data and (Fig. 10c) (Liu, M. et al. 2022a). The design structure linked with BOT is a combination of a CNN, an MLP, and a neural network that makes decisions. The task of the CNN in this structure is to generate a 512-dimensional feature vector that corresponds with pressure data. Conversely, the multilayer neural network is designed to create a 100-dimensional feature vector that aligns with olfactory data. These two feature vectors are then merged by the decision neural network into a 612-dimensional feature vector, which then carries out the associated learning to accomplish high-precision object recognition. This process corroborates the assertion that merging multimodal data from various sensor types and machine learning algorithms can lead to the development of a high-accuracy, tolerant learning system. Such a system is well-suited for tasks involving high-precision object recognition and decision-making in complex environments. Figure 10d shows a minimalist, low-power, and multimodal noncontact HMIs by combining a MEMS humidity sensor and a triboelectric sensor (Le et al. 2022). Current noncontact HMIs grapple with challenges including excessive power consumption, complicated signal processing circuits, and an absence of support for multidimensional interaction. However, this new HMI overcomes these problems by fusing complementary information from both sensors. The MEMS humidity sensor, featuring an aluminum nitride (AlN) bulk wave resonator and a graphene oxide (GO) film, is ultra-tiny and has high signal strength and low signal noise levels, which allows for continuous and stable interaction with a finger as it approaches. The triboelectric sensor, composed of two annular aluminum electrodes, promptly identifies finger movements in multiple directions. By combining these sensors, the proposed interface can be used as a game control interface for VR and a password input interface for high-security 3D passwords, showing its potential for a myriad of applications in the forthcoming Metaverse. The humidity sensor delivers high sensitivity, low signal noise, exceptional repeatability, and swift response and recovery speed, while the triboelectric sensor provides supplementary tracking of multidirectional and dynamic finger movements. Future applications could permeate into wearable domains in the Metaverse, employing flexible or textile substrates as the foundational platform.

Moreover, Shi. et al. developed a soft robotic perception system that integrates an ultrasonic sensor with flexible triboelectric sensors to achieve remote object positioning and multimodal cognition (Fig. 10e) (Shi et al. 2023). This system tackles the limitations tied to traditional guidance systems that rely on cameras or optical sensors, including challenges such as poor adaptability to varying environments, elevated data complexity, and cost inefficiencies. Employing reflected ultrasound, the ultrasonic sensor discerns the shape and distance of objects, thus empowering the robotic manipulator to orient itself suitably for object capture. As the grasping phase unfolds, a combination of ultrasonic and triboelectric sensors collects diverse sensory data, such as the object's upper contour, dimensions, form, rigidity, and material constitution. This data is subsequently merged and analyzed via deep learning, resulting in a substantial enhancement in object identification accuracy (up to 100%). The proposed perception system offers a straightforward, cost-effective, and efficient strategy for amalgamating positioning capabilities with multimodal cognitive intelligence in soft robotics. It notably broadens the functionalities and adaptabilities of current soft robotic systems in industrial, commercial, and consumer applications, demonstrating immense potential for automatic sorting, unmanned shops, and healthcare assistance in the age of IoT and AI.

## AI-enhanced self-sustainable system

**Fig. 11:**
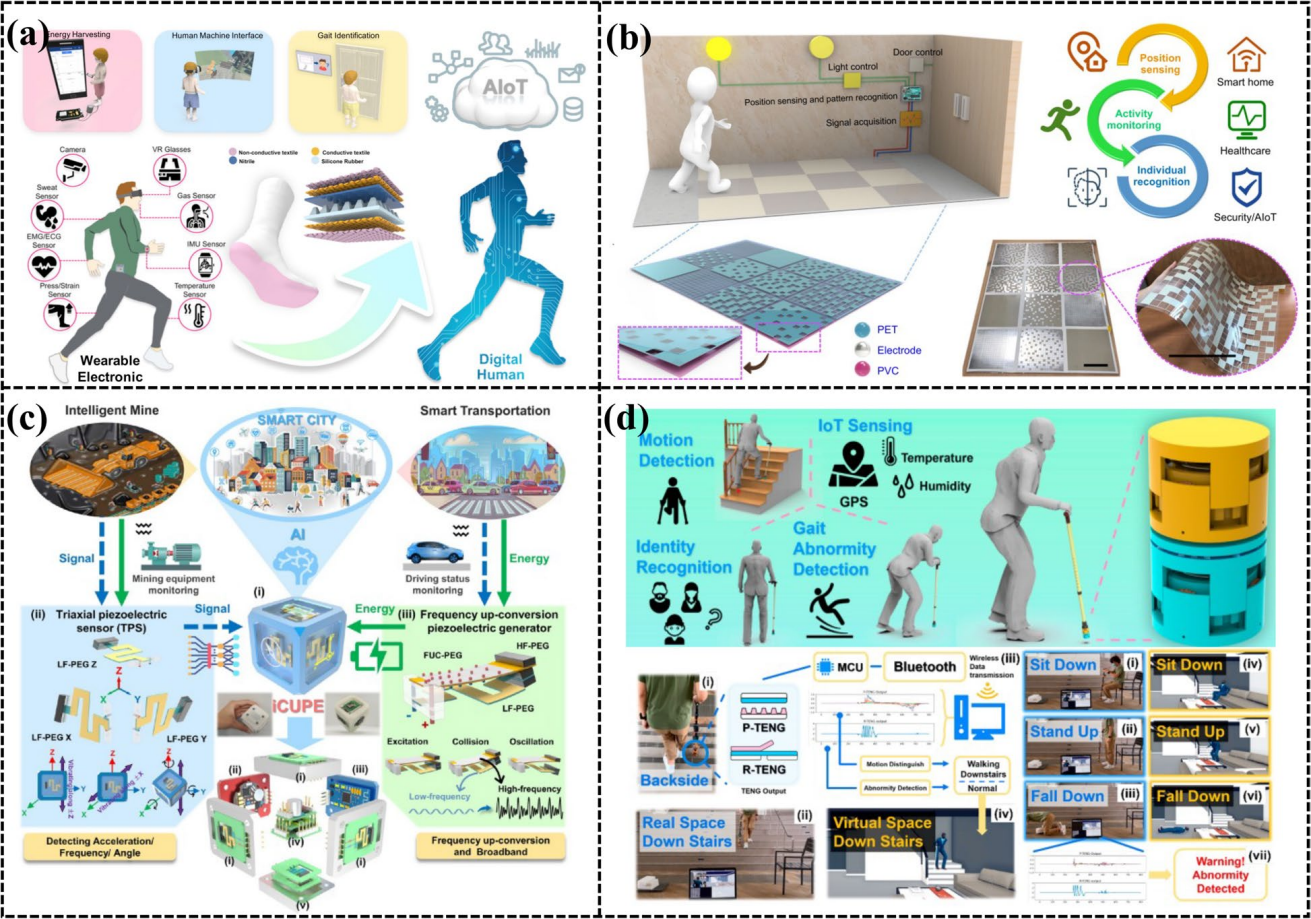
AI-enhanced self-sustainable system for Artificial Intelligence of Things (AIoT) applications. **a** A low-cost triboelectric sock equipped with the function of energy harvesting and gait sensing (Zhang, Z. et al., 2020b). **b** A deep learning-enabled smart mat capable of position/activity sensing and individual recognition (Shi, Q. et al. 2020b). **c** A self-powered piezoelectric AIoT node for smart mining, factory automation, transportation, and smart city applications (Huang et al. 2023). **d** A multifunctional walking stick for the care of the elderly (Guo, X. et al. 2021b)

In addition to glove sensors, the sensors attached to the foot can provide valuable information for healthcare and personal identification applications. TENG-based devices on foot can combine the functions of energy harvesting and gait analysis to create a self-powered system (Aqueveque et al. 2020; Han et al. 2019). While insoles have been commonly used for energy harvesting and gait sensing, socks may provide a more comfortable and flexible alternative, particularly for indoor scenarios where shoes are not worn. As shown in Fig. 11a, Zhang et al. developed smart socks based on TENG, which are designed not only to capture and repurpose waste energy generated by low-frequency body movements but also to transmit sensory data wirelessly (Zhang, Z. et al. 2020). Furthermore, these wearable sensors can collect and provide crucial information regarding the wearer's identity, health status, and activity level. The raw data collected during a dynamic gait cycle is fed directly into a 1D CNN model for training, without complicated pre-processing steps. Equipped with a multi-pixel sensor array, the smart sock has the capacity to accumulate extensive information from foot movements, enabling sophisticated gait analysis and potentially boosting accuracy across a broader cohort of users. A high accuracy of 93.54% is achieved when identifying 13 individual gait patterns. By employing deep-learning analysis, the smart socks have the potential to foster safer and smarter environments in smart buildings, eliminating the need for cameras and microphones.

Beyond the use of smart socks for gait analysis, another common technique involves using floor mat-based sensors. Shi et al. propose a deep learning-enabled smart mat (DLES-mat) array capable of position/activity sensing and individual recognition, as shown in Fig. 11b (Shi, Q. et al. 2020b). Since each person's walking gait patterns are unique, distinctive output voltage patterns are produced when a person walks on the triboelectric DLES-mat array. As an individual traverses the DLES-mat array, periodic contact-separation motions from human steps generate triboelectric output signals on the two sensing electrodes. The CNN-based deep learning model achieves a high recognition performance with an accuracy of 96.0%. The trained model holds significant potential for real-time control based on the identity recognition of similar gait patterns. The DLES-mat is portrayed as a digital twin system in a virtual corridor environment that replicates a real corridor, reflecting the real-time status of users. This encompasses position sensing via peak detection and individual recognition through deep learning prediction. Contrasting with camera-based monitoring systems that often provoke privacy concerns, this smart floor monitoring system employs a digital twin of the individual in a virtual setting, displaying solely the position information and recognized identity, which is crucial for automation, healthcare, and security applications.

Moreover, Huang et al. a self-powered piezoelectric AIoT node, named iCUPE (Fig. 11c) (Huang et al. 2023). Built around a three-dimensional (3D) hexahedral modular arrangement, the iCUPE embodies a self-sustainable system with AIoT capabilities. It incorporates six interchangeable modules, each serving a unique function and strategically positioned on its six faces. These modules include those for sensing temperature and humidity, a Bluetooth communication module, a central unit for processing data, and a frequency up-conversion piezoelectric generator (FUC-PEG) module. The role of the FUC-PEG module is to widen the frequency bandwidth of the iCUPE, achieved by uniting a low-frequency piezoelectric generator (LF-PEG) with a thick-film high-frequency piezoelectric generator (HF-PEG). This module transforms low-frequency inputs into high-frequency self-oscillations, thus generating an open-circuit voltage of 48 V under conditions of low frequency. Remarkably, the iCUPE, without requiring an external power supply, is competent at capturing vibrational signals in its environment, thereby yielding preliminary data points such as frequency and acceleration. A self-powered triaxial piezoelectric sensor (TPS) combined with machine learning integrates three orthogonal piezoelectric sensing units using low-frequency piezoelectric generators (LF-PEGs). This integration enables high-precision, multifunctional vibration recognition with resolutions of 0.01g for acceleration, 0.01 Hz for frequency, and 2° for tilting angle. Consequently, the TPS can achieve high recognition accuracy, ranging from 98% to 100%.

In response to the needs of the global elderly population and those with mobility impairments - a demographic that has already surpassed one billion and continues to rise - a multifunctional walking stick is proposed, as depicted in Fig. 11d (Guo, X. et al. 2021b). This innovative walking stick is comprised of two main functional units: the hybridized unit and the rotational unit. The hybrid module, with a top press TENG (P-TENG), mid-positioned EMG, and bottom rotational TENG (R-TENG), tracks pressure dynamics. The P-TENG, layered with aluminum, nitrile, and Ecoflex, generates variable voltages based on applied pressure. Its bottom aluminum layer, split into five electrodes, records the walking stick's entire interaction with the ground, from contact to release. Analyzing the P-TENG output through a 1D-CNN deep learning structure, the stick identifies five distinct movements (rising, sitting, walking, ascending, and descending stairs), evaluates three separate statuses, and recognizes ten different users. The R-TENG, on the other hand, flags unusual gait patterns, like falls, by monitoring output signals. An AI-driven virtual environment, designed to mimic real-world scenarios, accurately represents the user's real-time motion. The P-TENG and R-TENG's output signals, collected by an MCU module, are wirelessly sent to a computer for interpretation. The deep learning model captures the user's live motion status at home and mirrors it in the virtual environment. If a fall occurs, the anomaly is detected immediately, allowing for a quick call for help. Different from traditional indoor health surveillance systems, this walking stick prioritizes privacy by focusing solely on the user's motion status as a health indicator. Its design captures the ultra-low-frequency movement of those with mobility impairments, converting low-frequency linear motion into high-speed rotation. The mechanical energy harvested powers a self-sustaining IoT system with GPS tracking, and environmental temperature and humidity sensing capabilities, ensuring comprehensive user monitoring. This innovative integration of AIoT makes the walking stick an effective tool for mobility and health monitoring.

## Conclusion

**Fig. 12:**
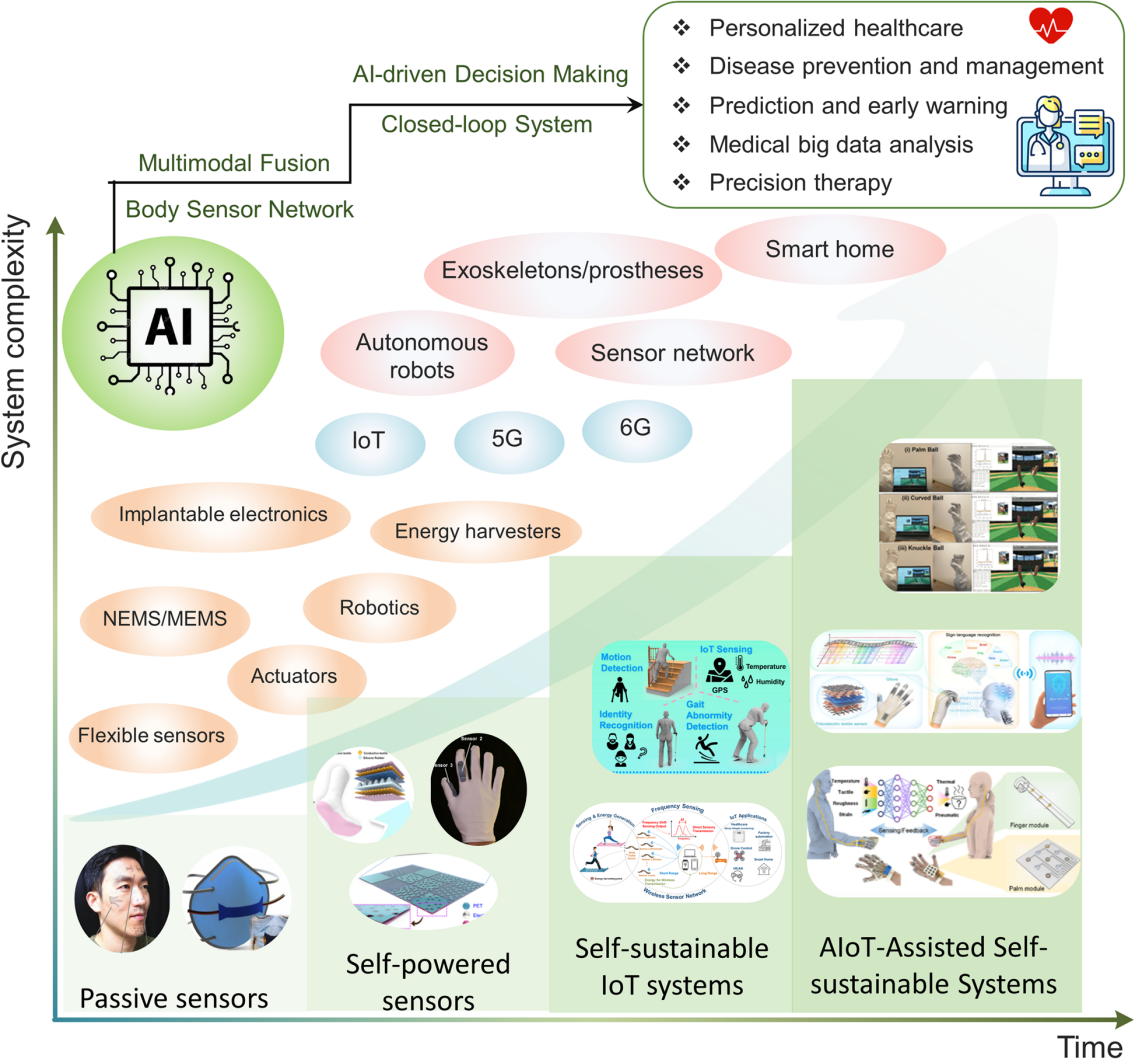
An overview of technology evolution from discrete passive sensors to comprehensive and capable AIoT-assisted self-sustainable systems for diversified healthcare and biomedicine applications (Clevenger et al. 2021; Guo, X. et al. 2021b; He et al. 2019; Shi, Qiongfeng et al. 2020b; Shin et al. 2020; Wen, Feng et al. 2020b; Wen, Feng et al. 2020c; Wen, Feng et al. 2021; Zhang, Z. et al. 2020; Zhu et al. 2022)

In this review article, we have explored the advancements and potential of AI-enhanced sensors in enabling next-generation healthcare and biomedical platforms, as shown in Fig. 12. Over the past few decades, there has been significant progress in the development of flexible sensors, which have demonstrated remarkable capabilities in reliably monitoring vital biomedical and physiological data, as well as daily activities. Concurrently, the emergence of energy harvesters and self-powered sensors has presented promising alternatives and supplementary approaches in establishing a bodyNET framework. This framework holds the potential to create self-sustaining sensing systems characterized by energy autonomy and extended operational lifespans. Meanwhile, notable advancements in the fields of flexible electronics and MEMS/ NEMS have fostered the progress of implantable devices for sensing, neural signal recording/modulation, and actuation. Moreover, with the advent of advanced wireless communication technologies such as 5G and 6G, alongside the integration of IoT devices and AI technology, an interconnected network known as the AIoT becomes feasible. This network, coupled with a cloud server infrastructure, facilitates the systematic collection, storage, and analysis of data, enabling intelligent decision-making processes. By leveraging the ongoing advancements in materials, devices, and systematic integrations, the vision of a comprehensive body sensor network comprising sensors both inside and on the body, as well as closed-loop systems, is within reach.

The integration of AI into sensor technologies holds immense promise for the future of healthcare and biomedicine. One of the key benefits of AI-enhanced sensor systems is the realization of personalized healthcare. By continuously monitoring physiological parameters and seamlessly integrating them with an individual's medical history, AI algorithms can generate personalized recommendations for disease prevention, early intervention, and chronic disease management. In addition, AI-enabled sensors can facilitate real-time monitoring of vital signs, enabling rapid detection of abnormalities and timely intervention, thus potentially saving lives. Empowered by advanced therapeutic devices, such as drug delivery patches and neural interfaces, closed-loop sensing-therapy systems emerge as a promising platform to provide enhanced benefits to patients suffering from chronic diseases. Looking ahead, the future of AI-enhanced sensors in healthcare and biomedicine appears even more promising. The convergence of AI with self-sustainable IoT systems will enable continuous and pervasive monitoring of an individual's health status, both inside and outside the body. The seamless integration of sensors, AI algorithms, and wireless communication technologies will empower patients to actively participate in their healthcare management, fostering a proactive approach to well-being. Additionally, the widespread adoption of AI in medical research and clinical practice will promote the development of advanced therapeutics and precision medicine tailored to individual patients. However, the successful implementation of AI-enhanced sensors in healthcare and biomedicine also raises several challenges that should be carefully addressed. Ensuring data privacy and security, addressing regulatory and ethical considerations, and developing robust validation frameworks are critical for building trust in AI-assisted systems. As AI-enhanced sensors collect and process vast amounts of personal health information, robust measures must be in place to safeguard patient privacy and prevent unauthorized access or data breaches. Given the role of these sensors in making critical healthcare decisions, compliance with regulatory frameworks becomes even more crucial. Thorough validation processes are also imperative in establishing a reliable and effective AI-assisted healthcare and biomedical system. They should involve rigorous testing against established standards, comparison with existing clinical practices, and verification of performance across diverse patient populations. Validation should encompass not only the accuracy of AI algorithms but also their generalizability, robustness to variations in data, and interpretability. Furthermore, continued advancements in materials, miniaturization, power management, and wireless communication will be essential to realize the full potential of AI-enhanced sensors.

## Data Availability

Not applicable.

## Abbreviations

AI: Artificial intelligence
HMI: Human-machine interfaces
AIOT: Artificial intelligence of things
IOT: Internet of Things
VOC: Volatile organic compounds
ML: Machine learning
